# Phylogenetic position and plastid genome structure of *Vietorchis*, a mycoheterotrophic genus of Orchidaceae (subtribe Orchidinae) endemic to Vietnam

**DOI:** 10.3389/fpls.2024.1393225

**Published:** 2024-05-24

**Authors:** Tahir H. Samigullin, Maria D. Logacheva, Leonid V. Averyanov, Si-Jin Zeng, Long-Fei Fu, Maxim S. Nuraliev

**Affiliations:** ^1^ A.N. Belozersky Institute of Physico-Chemical Biology, M.V. Lomonosov Moscow State University, Moscow, Russia; ^2^ Center for Molecular and Cellular Biology, Skolkovo Institute of Science and Technology, Moscow, Russia; ^3^ Komarov Botanical Institute of the Russian Academy of Sciences, St. Petersburg, Russia; ^4^ State Key Laboratory of Plant Diversity and Specialty Crops / Key Laboratory of Plant Resources Conservation and Sustainable Utilization, South China Botanical Garden, Chinese Academy of Sciences, Guangzhou, China and South China National Botanical Garden, Guangzhou, China; ^5^ Guangxi Key Laboratory of Plant Conservation and Restoration Ecology in Karst Terrain, Guangxi Institute of Botany, Guangxi Zhuang Autonomous Region and Chinese Academy of Sciences, Guilin, China; ^6^ Department of Higher Plants, Faculty of Biology, M.V. Lomonosov Moscow State University, Moscow, Russia; ^7^ Joint Russian-Vietnamese Tropical Scientific and Technological Center, Hanoi, Vietnam

**Keywords:** genome reductive evolution, non-photosynthetic plants, *Silvorchis*, *Sirindhornia*, taxonomy, tribe Orchideae

## Abstract

The orchid genus *Vietorchis* comprises three species, all discovered in the 21 century. Each of these species is achlorophyllous, mycoheterotrophic and is known to be endemic to Vietnam. The type species of the genus, *V. aurea*, occurs in a single location in northern Vietnam within a lowland limestone karstic area. *Vietorchis furcata* and *V. proboscidea*, in contrast, are confined to mountains of southern Vietnam, far away from any limestone formations. Taxonomic placement of *Vietorchis* remained uncertain for the reason of inconclusive morphological affinities. At the same time, the genus has never been included into molecular phylogenetic studies. We investigate the phylogenetic relationships of two species of *Vietorchis* (*V. aurea* and *V. furcata*) based on three DNA datasets: (1) a dataset comprising two nuclear regions, (2) a dataset comprising two plastid regions, and (3) a dataset employing data on the entire plastid genomes. Our phylogenetic reconstructions support the placement of *Vietorchis* into the subtribe Orchidinae (tribe Orchideae, subfamily Orchidoideae). This leads to a conclusion that the previously highlighted similarities in the rhizome morphology between *Vietorchis* and certain mycoheterotrophic genera of the subfamilies Epidendroideae and Vanilloideae are examples of a convergence. *Vietorchis* is deeply nested within Orchidinae, and therefore the subtribe Vietorchidinae is to be treated as a synonym of Orchidinae. In the obtained phylogenetic reconstructions, *Vietorchis* is sister to the photosynthetic genus *Sirindhornia*. *Sirindhornia* is restricted to limestone mountains, which allows to speculate that association with limestone karst is plesiomorphic for *Vietorchis*. Flower morphology is concordant with the molecular data in placing *Vietorchis* into Orchidinae and strongly supports the assignment of the genus to one of the two major clades within this subtribe. Within this clade, however, *Vietorchis* shows no close structural similarity with any of its genera; in particular, the proximity between *Vietorchis* and *Sirindhornia* has never been proposed. Finally, we assembled the plastid genome of *V. furcata*, which is 65969 bp long and contains 45 unique genes, being one of the most reduced plastomes in the subfamily Orchidoideae. The plastome of *Vietorchis* lacks any rearrangements in comparison with the closest studied autotrophic species, and possesses substantially contracted inverted repeats. No signs of positive selection acting on the protein-coding plastid sequences were detected.

## Introduction

The genus *Vietorchis* Aver. & Averyanova was established to accommodate a newly described non-photosynthetic (presumably mycoheterotrophic) species, *V. aurea* Aver. & Averyanova, which appeared to be evidently distinct from all the other known genera of Orchidaceae (Averyanov and Averyanova, 2003). Ten years after the publication of the genus, its second species, *V. furcata* Aver. & Nuraliev, was described (Averyanov et al., 2013; see also Averyanov, 2013; Nuraliev et al., 2019). After another ten years, the third species of the genus, *V. proboscidea* Aver., Vuong & V.C.Nguyen, was introduced, which is extremely close morphologically to *V. furcata* (Averyanov et al., 2023). All three species of *Vietorchis* are currently known to be endemic to Vietnam. Of them, *V. aurea* is found only in Cuc Phuong National Park in the northern part of the country, whereas the other two species are confined to the southern part. *Vietorchis furcata* was reported from Chu Yang Sin National Park, Bao Loc forest and Hon Ba Nature Reserve, and *V. proboscidea* occurs in Dam Rong District, being nearly sympatric with *V. furcata*. The only known population of *V. aurea* is located within a vast limestone karst area (Tuan, 2020), where it inhabits lowland valley forest between rocky limestone hills (Averyanov, 2010). The other two species occur in mountainous areas devoid of limestone karstic formations.

Phylogenetic relationships and taxonomic placement of *Vietorchis* became a matter of continuous debates. Similarly to many other fully heterotrophic angiosperms, *Vietorchis* shows highly specialized morphology of both floral and underground parts, which complicates direct comparison with the proposed relatives. Initially *Vietorchis* was placed into the subfamily Orchidoideae, tribe Orchideae, subtribe Orchidinae (Averyanov and Averyanova, 2003). This placement was maintained by Averyanov (2010) who also indicated that *Vietorchis* is most close to *Silvorchis* J.J.Sm. *Silvorchis* is a poorly known Asian mycoheterotrophic genus; its type species, *S. colorata* J.J.Sm., was collected only once in 1907 in Java and is now probably extinct, and its second species, *S. vietnamica* Aver., Dinh & K.S.Nguyen, was recently discovered in Vietnam (Averyanov et al., 2018). Averyanov consistently accepted the subtribe Orchidinae in its narrow sense, i.e. separately from the subtribe Habenariinae (recognized e.g. in Averyanov, 2008; see also Averyanov et al., 2018) or Gymnadeniinae (recognized e.g. in Averyanov, 2010).

Averyanov et al. (2013) along with the description of *V. furcata* introduced a new subtribe, Vietorchidinae (within the tribe Orchideae), containing the genera *Vietorchis* and *Silvorchis*. Averyanov et al. (2013, 2018) have also provided a review of the opinions of various researchers on the affinities of *Silvorchis*; some taxonomists assumed this genus to be related to various representatives of the subfamily Orchidoideae, while the others argued for its relationship with the mycoheterotrophic genera *Epipogium* J.G.Gmel. ex Borkh. and *Stereosandra* Blume within the subfamily Epidendroideae. Meanwhile, Orchidoideae and Epidendroideae are the most diverse subfamilies of Orchidaceae. According to modern phylogenetic views (Chase et al., 2015), they crown the orchid evolution forming the terminal branch of a grade. The rest of the grade is formed by the subfamilies Cypripedioideae, Vanilloideae and the basalmost Apostasioideae, which altogether comprise about 1% of the species diversity of Orchidaceae. The striking contradictions regarding the relationships of *Silvorchis*, which is morphologically similar to *Vietorchis*, is a consequence of discrepancy in structure of above-ground and underground organs of these plants. Gynostemium and pollinaria of *Silvorchis* and *Vietorchis* are similar to those of some Orchidinae, for example *Brachycorythis* Lindl. and *Orchis* L. (*Platanthera* Rich. was erroneously mentioned by Averyanov et al., 2013). At the same time, the fleshy rootless rhizomes (described as tuberoid rhizomes and rhizome-like tubers) make them close to several mycoheterotrophic lineages belonging to Epidendroideae (*Epipogium*, *Gastrodia* R.Br., *Yoania* Maxim.), Vanilloideae (*Cyrtosia* Blume, *Galeola* Lour., *Lecanorchis* Blume) and *Odontochilus* Blume from Orchidoideae-Cranichideae-Goodyerinae (Averyanov et al., 2013, 2018).

In most recent accounts, the preference is given to the flower structure, and the placement of *Vietorchis* and *Silvorchis* in the subfamily Orchidoideae is accepted (e.g. Chase et al., 2015). Averyanov et al. (2018) maintained them in the subtribe Vietorchidinae. Olędrzyńska et al. (2016) synonymized Vietorchidinae with Orchidinae but provided no explanation in favor of their views. Averyanov et al. (2013) argued that the non-typical rhizome morphology could evolve within Orchidoideae in the course of adaptation to the mycoheterotrophic mode of life. However, the precise relationships of these genera cannot be confidently established on the basis of morphological features alone. As pointed by Chase et al. (2015), molecular data are needed to elucidate placement of these two genera within the taxonomic system of Orchidaceae.

Suggestions to merge the genus *Vietorchis* within *Silvorchis* were proposed, and corresponding nomenclatural combinations, *Silvorchis aurea* (Aver. & Averyanova) Szlach. and *S. furcata* (Aver. & Nuraliev) Olędrz. & Szlach., were published (Szlachetko et al., 2006; Olędrzyńska et al., 2016; see also Olędrzyńska and Szlachetko, 2021). These taxonomic transfers, however, were not accompanied by any additional data on these plants, and lack sufficient substantiations for the corresponding decisions. Besides, the synonymization of *Vietorchis* with *Silvorchis* leads to a loss of taxonomic information: the species within each of these genera are clearly highly similar to each other, whereas the similarity between the genera is not so high. This would be neglected if a single genus (containing five species) is accepted. For this reason, we prefer to consider *Vietorchis* a distinct genus, even though it is treated, rather groundlessly, as a synonym by Chase et al. (2015), Govaerts et al. (2022) and Wei et al. (2022).

Neither *Silvorchis* nor *Vietorchis* have ever been included into a molecular phylogenetic analysis. While no material of *Silvorchis* is currently available for such a study, our material of *Vietorchis aurea* and *V. furcata* allows a comprehensive DNA investigation, which is performed here in order to clarify the phylogenetic relationships of this genus as well as evolution of key morphological features in this group of Orchidaceae. We present phylogenetic reconstructions based on three datasets: (1) a dataset comprising selected nuclear regions, (2) a dataset comprising selected plastid regions, and (3) a dataset employing data on the entire plastid genomes. The two plastid datasets differ in taxonomic sampling and in number of molecular markers. Since an adequate taxon sampling is crucial for correct phylogeny reconstruction, we compiled a dataset representing the main lineages and genera of the subtribe Orchidinae s.l. (i.e., sensu Jin et al., 2017) using two plastid markers. At the same time, employment of longer matrices of complete plastome data allows to reduce stochastic error in phylogeny estimation; therefore, we sequenced plastid genomes of the two species of *Vietorchis* and used the obtained sequences in the dataset of complete plastomes, which was less representative in terms of species sampling. This approach allows more confident phylogenetic conclusions: similar results obtained from different datasets would indicate a robustly supported reconstruction.

Apart from the resolution of the phylogenetic questions, data on plastome of *Vietorchis* are important for understanding of plastid evolution in heterotrophic higher plants. Transitions from autotrophy to heterotrophy are usually accompanied by substantial structural changes of plastid genomes that lead to plastome reductions (Barrett and Davis, 2012; Barrett et al., 2014; Wicke et al., 2016; Graham et al., 2017), sometimes to the drastic ones, with the extreme known cases being those of *Pilostyles* Guill. from Apodanthaceae (Arias-Agudelo et al., 2019) and *Pogoniopsis* Rchb.f. from Orchidaceae (Klimpert et al., 2022). It is therefore of special interest if the plastome of *Vietorchis* shares the major trends of the nonphotosynthetic plant plastomes. Here we report for the first time the structure of the plastid genome in *Vietorchis*, accompanied by its comparative analysis.

## Materials and methods

### Plastid genome of *Vietorchis*: sequencing, assembly and comparative analyses

Total genomic DNAs were extracted from herbarium material (*V. aurea*) and silica gel-dried material (*V. furcata*) using the CTAB-based method (Doyle and Doyle, 1987) with the following modifications: chloroform extraction was performed twice. DNA of *V. furcata* was additionally extracted using the DiamondDNA kit (DiamondDNA, Russia) for clarification of the borders of the inverted repeat (IR) and single copy (SC) regions. For library preparation, we used NEBNext Ultra II DNA sample preparation kit for Illumina (New England Biolabs, USA). Before processing, DNA was sheared using Covaris S220 sonicator (Covaris, USA) with the following settings: time 40 s, peak power 175 W, duty cycle 10%. Libraries were sequenced using Hiseq2000 (*V. aurea*) or Nextseq (*V. furcata*) instruments (Illumina, USA).

We failed to combine a complete plastome of *V. aurea* as only short non-overlapping plastid contigs were assembled; nevertheless, these data were useful for employment in the phylogenetic analyses.

For the plastome of *V. furcata*, *de novo* assembly was performed using a CLC Genomics Workbench and IDBA version 1.1.3 (Peng et al., 2012). Contigs showing similarity to plastid genomes were joined by overlapping ends. To check the accuracy of assembly, trimmed paired reads were mapped onto the assembled plastome sequence and the mapping was examined in order to check that there are no regions with gaps in coverage (see **Supplementary Figure 1** for the borders of inverted repeats); also, PCR and Sanger sequencing were used for the verification of IR-SC borders. The following primers flanking the IR-SC borders were used: Vf35037F: ATTTCGATTAGGGTCGTATTCTATGG, Vf35269R: CACGGCAATACATTTATACAAAACTTC; Vf35970F: TTCGTGGATCAATTTTAATTCAGTGG, Vf36190R: ATGAAAATATTCGCGATACTTGGTTG. PCR was run on T100 Thermal Cycler (Bio-Rad, USA) using Encyclo PCR kit (Evrogen, Russia) under the following program: initial denaturation for 3 min at 95°C, followed by 35 cycles each comprising 15 s at 95°C, 25 s at 58°C and 40 s at 72°C. PCR products were visualized on agarose gel, cleaned using AMPure beads (Beckman Coulter, USA) and submitted for sequencing to “Genome” sequencing facility (Engelhardt Institute of Molecular Biology of the Russian Academy of Sciences). Sequencing reaction was performed using BigDye Terminator v. 3.1 kit (Thermo Fisher Scientific, USA) and run on a sequencing instrument Applied Biosystems 3730 DNA Analyzer (Thermo Fisher Scientific, USA).

Annotation of plastid genes was performed using the GeSeq web tool (Tillich et al., 2017) with land plant plastid sequences as a reference set.

Colinearity of the sampled plastomes was estimated using the Mauve program (Darling et al., 2004).

Dispersed repeat content was explored using the repeat finder module in the Unipro UGENE package version 37.0 (Okonechnikov et al., 2012) with minimal length restricted to 20 bases for direct, inverted and palindrome repeats.

Estimation and comparison of synonymous and nonsynonymous substitution rates in *V. furcata* and other orchid lineages were performed using the CodeML program from PAML package (Yang, 1997, 2007) with EasyCodeML interface (Gao et al., 2019). The tree inferred from the phylogenetic analysis of the 29-gene set was used as the input tree. A hypothesis that natural selection acting on the plastid proteins of *V. furcata* (25 of which were revealed in this study, see below) differs from those of other orchids was tested using two branch models, assuming a single omega value (ω, nonsynonymous to synonymous substitution rate ratio) for all branches versus different values of omega for the *V. furcata* branch (foreground) and the rest of the branches (background). Besides, the branch-site test was performed to detect signs of possible positive selection affecting a few sites of a protein in the *V. furcata* branch; Bonferroni correction was applied in both tests. Additionally, a similar gene-wide test for positive selection implemented in the BUSTED web tool (Murrell et al., 2015) was performed.

### Taxon sampling for phylogenetic analyses

**Figure 1:**
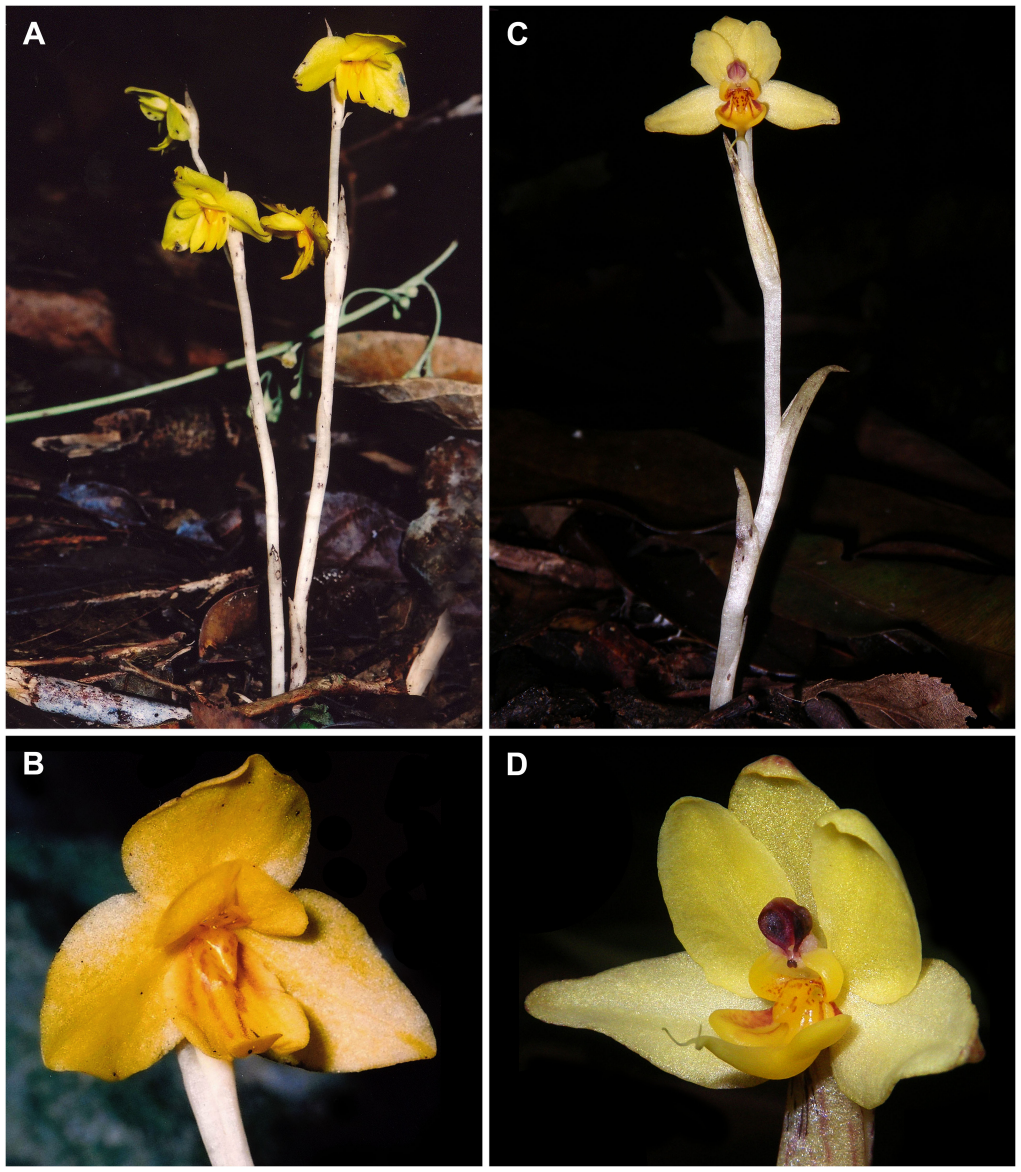
Studied species of *Vietorchis*. **(A, B)** *Vietorchis aurea*: habit and flower (*May Van Xinh MVX 261*; LE01076723, LE01122406). Photos by May Van Xinh. **(C, D)** *Vietorchis furcata*: habit (*Nuraliev 747*; LE01076758, LE01122414) and flower (*Nuraliev et al. 810*; LE01076727, LE01122413). Photos by M.S. Nuraliev.

**Figure 2:**
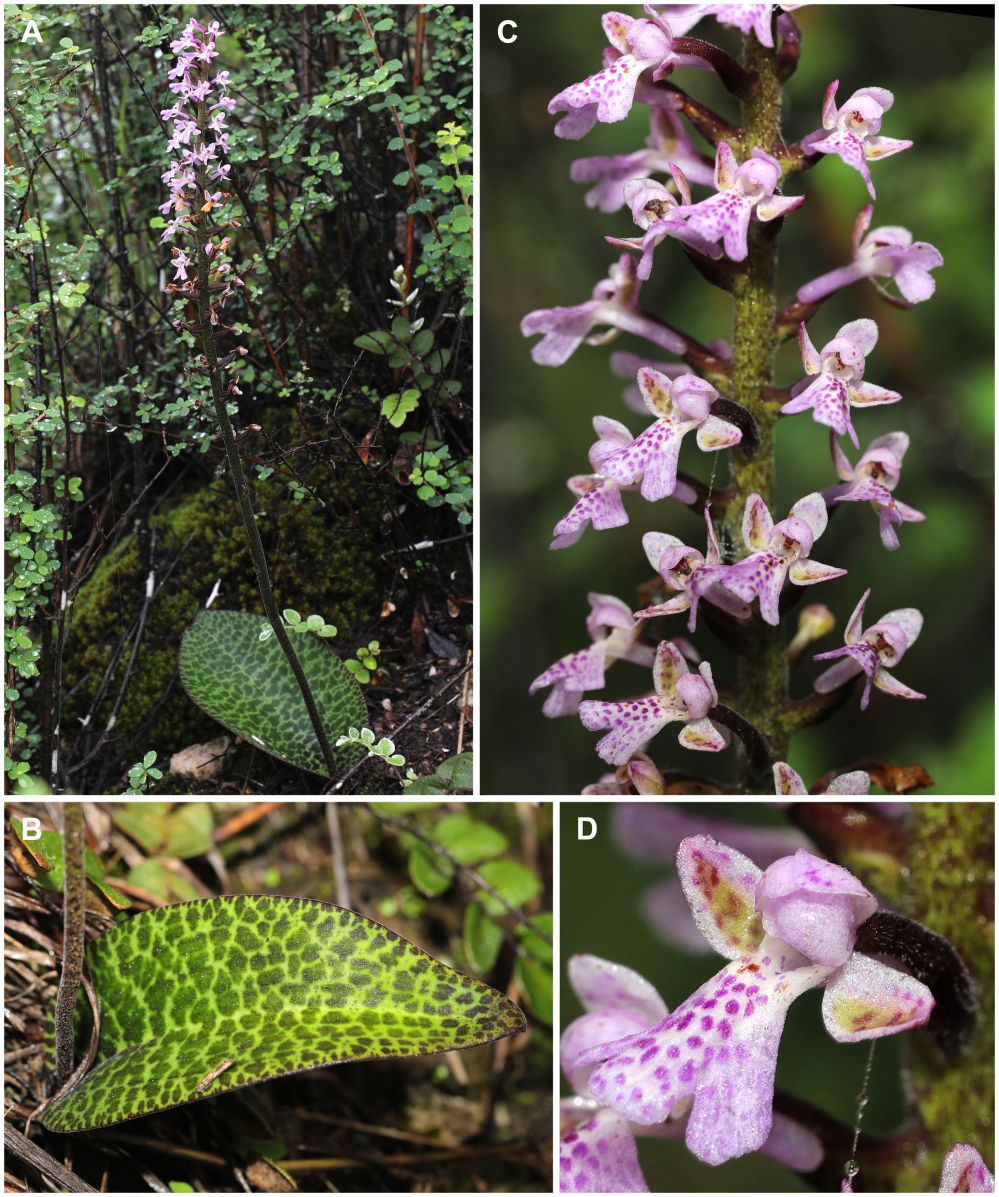
*Sirindhornia monophylla* in Yunnan, China (LE01093267). **(A)** Habit. **(B)** Leaf. **(C)** Inflorescence. **(D)** Flower. Photos by Qin-Chang Liao.

Sequences for *Vietorchis aurea* (**Figures 1A, B**) and *V. furcata* (**Figures 1C, D**) were generated *de novo*, representing the first DNA data obtained for this genus. In order to get rough estimates of the phylogenetic affinities of *Vietorchis*, the obtained ITS1–2 regions of both species were searched against the NCBI database using the BLAST tool. The analyses indicated high similarity with *Sirindhornia* H.A.Pedersen & Suksathan (93–95%) and the related genera from Orchidinae. Thus, in further phylogenetic analysis we focused on this subtribe. Additionally, sequences for *Sirindhornia monophylla* (Collett & Hemsl.) H.A.Pedersen & Suksathan (**Figure 2**) were generated for the first time. The rest of the sequences were obtained from GenBank. The outgroup taxa were selected based on Jin et al. (2017).

Our datasets employing selected nuclear and plastid DNA regions were based on the dataset for the subtribe Orchidinae used by Jin et al. (2017). We compiled a sampling of accessions following the idea that the generic diversity should be represented the best, as well as the basal lineages of the main clades. Fifty five specimens were employed in total (including those used by Jin et al., 2017 and studied here for the first time). These specimens belong to 54 species and 24 genera. Of them, *Disperis* sp., *Goodyera schlechtendaliana* Rchb.f. and *Spiranthes sinensis* (Pers.) Ames were included as outgroup taxa.

For the dataset based on the entire plastid genomes, all available published information (by March 30, 2023) on plastome structure of the species belonging to the subtribe Orchidinae was used. *Goodyera pubescens* (Willd.) R.Br. and *Spiranthes sinensis* were included as outgroup taxa. Altogether, this dataset covered 29 species (each represented by a single accession) and 13 genera.

Information on all the studied specimens is summarized in **Appendices A1** and **A2**. General taxonomy of Orchidaceae, including the generic placement of the studied species, follows Govaerts et al. (2022). In the Appendices, taxon names used in the cited papers are indicated in brackets for clarity, in case they are heterotypic synonyms of the currently accepted names, and also for the specimen of *Herminium tibeticum* X.H.Jin, Schuit. & Raskoti that was re-identified by Jin et al. (2017). The subtribe Orchidinae is accepted here in a wide sense, following Chase et al. (2015) and Jin et al. (2017), i.e. including the genera of the traditionally recognized subtribes Habenariinae and Satyriinae (e.g. Dressler, 1993; Bateman et al., 2003; Jin et al., 2012, Jin et al., 2014; Tang et al., 2015).

### Phylogenetic inference

Low-coverage genome sequencing that we used for the assembly of the plastid genes/genome of *Vietorchis* species also allowed us to assemble the nuclear ribosomal RNA operon. Its genes and spacers, especially the 18S gene and the spacers ITS1 and ITS2, are valuable as phylogenetic markers.

Within the datasets employing selected DNA regions, we used four markers: the nuclear ribosomal ITS1–2 region (including internal transcribed spacer 1, the 5.8S rRNA gene and internal transcribed spacer 2; together referred to as ITS), a part of the nuclear *Xdh* gene, a part of the plastid *matK* gene and the plastid *psb*A-*trn*H intergenic spacer region (including the *rps*19 gene). These regions have previously been successfully used for phylogenetic analysis of Orchidinae (Jin et al., 2014; Tang et al., 2015; Jin et al., 2017).

The plastid dataset was composed of rRNA and protein-coding genes (29 genes in total) found in the plastome of *V. furcata* (*accD*, *clpP*, *infA*, *matK*, *rpl14*, *rpl16*, *rpl2*, *rpl20*, *rpl22*, *rpl23*, *rpl32*, *rpl36*, *rps11*, *rps12*, *rps14*, *rps16*, *rps18*, *rps19*, *rps2*, *rps3*, *rps4*, *rps7*, *rps8*, *rrn16*, *rrn23*, *rrn4.5*, *rrn5*, *ycf1*, and *ycf2*). Sequences of the corresponding genes were manually extracted from 27 complete plastomes retrieved from Genbank. For *V. aurea*, partial sequences of 11 of these genes (*accD*, *clpP*, *infA*, *matK*, *rpl14*, *rpl16*, *rpl2*, *rps2*, *rps3*, *ycf1*, *ycf2*) were available from the obtained plastid contigs.

For phylogenetic purposes, the sequences of *Sirindhornia monophylla* were generated. Total genomic DNA was extracted from silica gel-dried leaves using a modified CTAB method (Smith et al., 1991). The primers used for amplification of the ITS, *Xdh* and *matK* regions are listed in **Table 1**. The *psb*A-*trn*H region was not investigated. For the PCR, we used MightyAmp DNA polymerase (Takara Bio Inc., Japan) with corresponding buffer in a 30 mkl reaction mix. The PCR program consisted of 38–42 cycles, with each cycle as follows: denaturation for 20–30 s at 98°C, primer annealing for 30 s at 53°C, and elongation for 60–120 s at 68°C, with an initial denaturation for 3 min at 98°C and the final extension for 7 min at 68°C. The PCR products were run on 1.5% agarose gels to check the quality of amplified DNA. Commercial service of purification and Sanger sequencing was provided by the Invitrogen (China). Both forward and reverse sequences were edited and assembled using DNASTAR (http://www.dnastar.com/).

**Table 1:** Primers used for amplification of the DNA regions of *Sirindhornia monophylla*.

Primer	Sequence (5’ to 3’)	Source
ITS-17SE	ACGAATTCATGGTCCGGTGAAGTGTTCG	Sun et al. (1994)
ITS-26SE	GAATTCCCCGGTTCGCTCGCCGTTAC	Sun et al. (1994)
*matK*-19F	CGTTCTCATATTGCACTATG	Molvray et al. (2000)
*matK*-713F	AAGAAAAGATTCTTTTGGTTCC	Liu et al. (2011)
*matK*-969R	CTTTTCCTTGATATCGAACAT	Liu et al. (2011)
*matK*-1867R	TTGCAGTTTTCATTGCACACG	Liu et al. (2011)
*Xdh*-590F	GTGAATTCATTTGCCCATCATCT	Hu et al. (2020)
*Xdh*-1513R	GAGTGCAATATCATCTTCTCTCCG	Hu et al. (2020)

For the specimens of *Vietorchis*, sequences of the regions ITS, *matK* and *psb*A-*trn*H were obtained from the results of the high-throughput sequencing (HTS, described above), but we were unable to get the *Xdh* sequences from the HTS data. We therefore used Sanger sequencing to obtain the *Xdh* sequence of one of the species, *V. furcata*. We used the following PCR primers: X502F (TGTGATGTCGATGTATGC), X1599R (G(AT)GAGAGAAA(CT)TGGAGCAAC), X551F (GAAGAGCAGATTGAAGA(AT)(AT)GCC) and X1591R (AA(CT)TGGAGCAACTCCACCA) (Górniak et al., 2010). The PCR program followed Górniak et al. (2010) and Jin et al. (2017). The PCR product was sent to the Majorbio Company (www.majorbio.com, China) for Sanger sequencing.

Sequences were aligned using MAFFT version 7.471 (Katoh et al., 2002; Katoh and Standley, 2013) and corrected manually in BioEdit (Hall, 1999). Regions where positional homology could not be firmly determined were excluded along with the gap-rich positions.

Phylogenetic reconstructions were performed for the concatenated alignments of the two nuclear markers (ITS+*Xdh*), for the concatenated alignments of the two plastid markers (*matK*+*psb*A-*trn*H), for the alignments of these four markers separately, and for the concatenated alignments of the 29 plastid genes.

The Bayesian phylogenetic reconstruction was performed by MrBayes v.3.2.7 (Ronquist et al., 2012) using four simultaneous runs of 20 million generations and four chains sampling every 1000th generation. The GTR+Γ model of nucleotide substitutions was selected for the data matrices (except for HKY+Γ for the *Xdh* set) as the most appropriate one according to the Akaike information criterion (Akaike, 1974) in PAUP version 4.0a (Swofford, 2003). The first million generations were discarded as burn-in, and the remaining trees were combined in a majority-rule consensus tree. Effective sample sizes were evaluated using Tracer v.1.7.1 (Rambaut et al., 2018). The effective sample sizes were > 200 for all statistics in all datasets, suggesting that the run length was adequate.

The maximum likelihood (ML) phylogenetic reconstruction was performed by IQ-tree (Minh et al., 2020). Internal branch support was assessed with the ultrafast bootstrap approximation (Hoang et al., 2018) using 10 thousand replications.

## Results

### Affinities of *Vietorchis* inferred from phylogenetic analysis

The main characteristics of the alignments are listed in **Table 2**. The Bayesian and ML approaches for each dataset resulted in generally congruent tree topologies (**Figures 3**–**5**; **Supplementary Figures 2–12**).

**Table 2:** Statistics of multiple alignments.

Marker(s)	Initial alignment length (bp)	Alignment length used in phylogeny inference (bp)	Number of variable sites used in phylogeny inference (bp)	Percent of variable sites used in phylogeny inference
*psb*A*-trn*H**	1159	751	202	26.9
*matK*	1911	1628	619	38
ITS	874	800	505	63.1
*Xdh*	931	928	398	42.8
plastome set	30671	28254	4943	17.5

**Figure 3:**
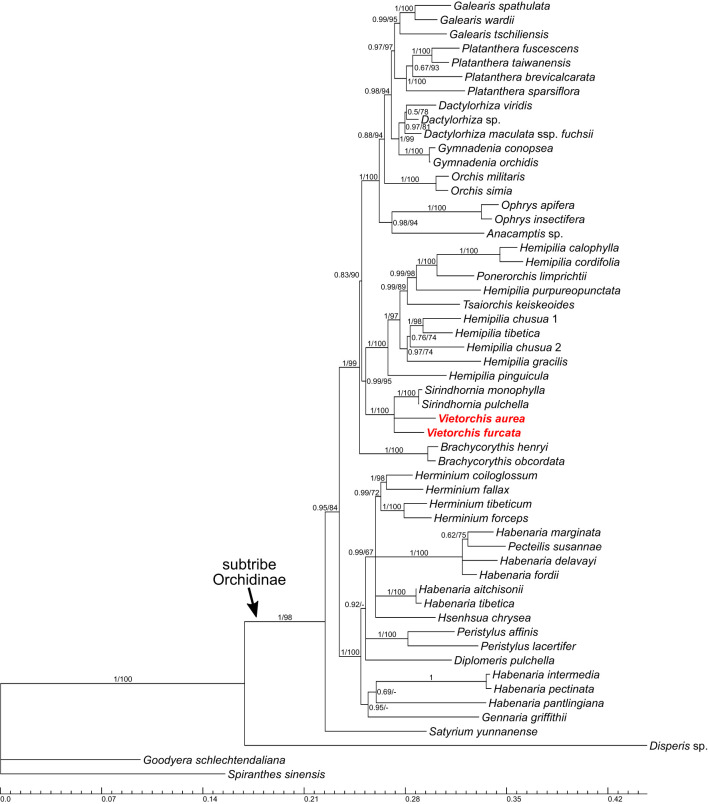
Phylogenetic tree obtained from the Bayesian analysis of the combined nuclear ITS+*Xdh* dataset. Numbers near branches indicate posterior probabilities (PP) / ultrafast bootstrap percentages in the maximum likelihood analysis (BP_ML_; see **Supplementary Figure 2**); “-” indicates absence of the branch in the maximum likelihood analysis. Scale bar shows number of substitutions per site. Accessions of *Vietorchis* are marked with red.

**Figure 4:**
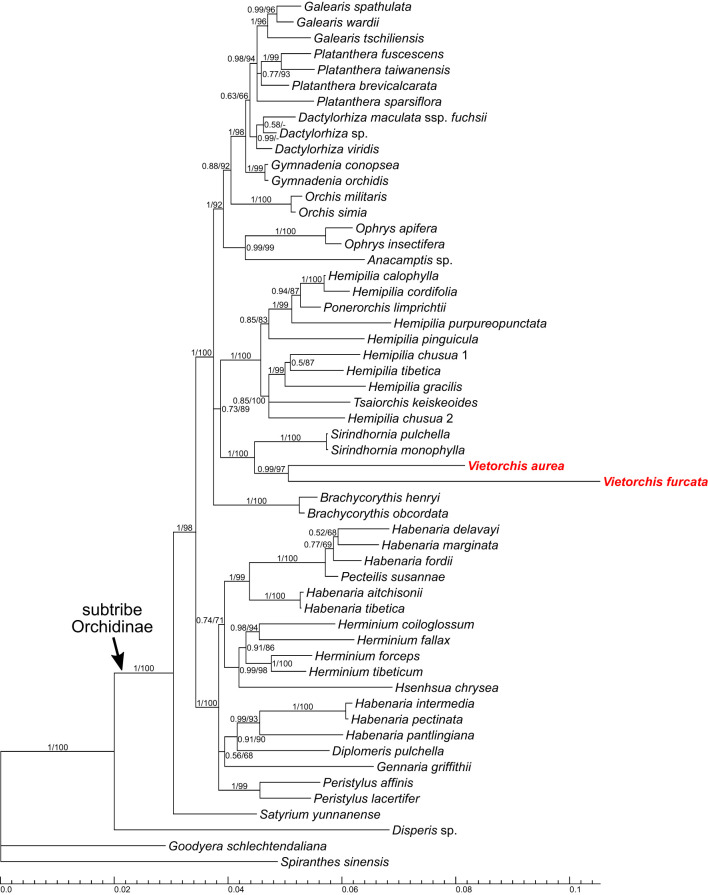
Phylogenetic tree obtained from the Bayesian analysis of the combined plastid *matK*+*psb*A-*trn*H dataset. Numbers near branches indicate posterior probabilities (PP) / ultrafast bootstrap percentages in the maximum likelihood analysis (BP_ML_; see **Supplementary Figure 3**); “-” indicates absence of the branch in the maximum likelihood analysis. Scale bar shows number of substitutions per site. Accessions of *Vietorchis* are marked with red.

**Figure 5:**
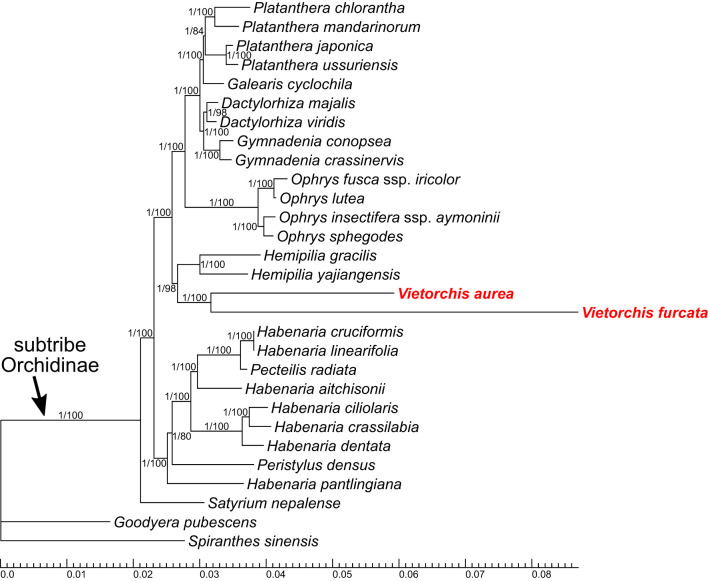
Phylogenetic tree obtained from the Bayesian analysis of the combined 29-gene plastid dataset. Numbers near branches indicate posterior probabilities (PP) / ultrafast bootstrap percentages in the maximum likelihood analysis (BP_ML_; see **Supplementary Figure 4**). Scale bar shows number of substitutions per site.

The analyses of four separate markers (ITS, *Xdh*, *matK*, *psb*A-*trn*H; **Supplementary Figures 5–12**) lack any well-supported incongruence with the two-marker trees (**Figures 3**, **4**; **Supplementary Figures 2, 3**), but only show a lower resolution.

In all the two-marker trees (i.e., the nuclear ITS+*Xdh* trees and the plastid *matK*+*psb*A-*trn*H trees), the genera belonging to the subtribe Orchidinae (as defined by Chase et al., 2015) form a well-supported clade [posterior probability (PP) 1.00 for both datasets, ultrafast bootstrap percentage in the maximum likelihood analysis (BP_ML_) 98 and 100, respectively]. The genus *Satyrium* occupies a basal position within Orchidinae (the monophyly of the rest of Orchidinae is supported with PP 0.95, BP_ML_ 84 for the nuclear dataset and PP 1.00, BP_ML_ 98 for the plastid dataset). The rest of the accessions form two clades sister to each other. One of them (PP 1.00, BP_ML_ 100 for both datasets) comprises all the species of *Habenaria* studied, intermixed with the species of *Diplomeris*, *Gennaria*, *Herminium*, *Hsenhsua*, *Pecteilis*, and *Peristylus*. The second clade (PP 1.00 for both datasets, BP_ML_ 99 and 100, respectively) is subdivided into three subclades. One of them is *Brachycorythis* (PP 1.00, BP_ML_ 100 for both datasets; *Brachycorythis* occupies a sister position to the rest of the clade in the nuclear trees, and forms a polytomy with the two other subclades in the plastid trees). The second subclade (PP 1.00 for both datasets, BP_ML_ 100 and 92, respectively) contains species of the mostly extra-tropical genera (*Anacamptis*, *Dactylorhiza*, *Galearis*, *Gymnadenia*, *Ophrys*, *Orchis*, *Platanthera*). In the third subclade (PP 0.99, BP_ML_ 95 for the nuclear dataset and PP 0.73, BP_ML_ 89 for the plastid dataset), a clade comprising *Sirindhornia* and *Vietorchis* (PP 1.00, BP_ML_ 100 for both datasets) is sister to a clade containing the species of *Hemipilia* together with *Ponerorchis limprichtii* and *Tsaiorchis keiskeoides* (PP 1.00, BP_ML_ 100 for both datasets), i.e. corresponding to *Hemipilia* sensu latissimo as accepted by Tang et al. (2015) and Yang et al. (2022). The clade comprising *Sirindhornia* and *Vietorchis* shows a tritomy in the nuclear trees with the following branches: *V. aurea*, *V. furcata* and the monophyletic *Sirindhornia* (*S. monophylla* + *S. pulchella*; PP 1.00, BP_ML_ 100). In the plastid trees, both *Sirindhornia* (PP 1.00, BP_ML_ 100) and *Vietorchis* (PP 0.99, BP_ML_ 97) are monophyletic.

The trees based on the 29-gene plastid dataset (**Figure 5**, **Supplementary Figure 4**) are highly congruent to the two-marker trees (ITS+*Xdh* and *matK*+*psb*A-*trn*H), despite it employed a smaller sampling that was quite different at the species level. In the 29-gene trees, the monophyly of the subtribe Orchidinae is supported (PP 1.00, BP_ML_ 100). *Satyrium* is the basalmost taxon within Orchidinae, and the other accessions are distributed within two clades. One of these clades comprises the species of *Habenaria* intermixed with *Pecteilis* and *Peristylus* (PP 1.00, BP_ML_ 100). Within the second clade (PP 1.00, BP_ML_ 100), the subclade containing species of the mostly extra-tropical genera (*Dactylorhiza*, *Galearis*, *Gymnadenia*, *Ophrys*, *Platanthera*; PP 1.00, BP_ML_ 100) is sister to a subclade (PP 1.00, BP_ML_ 98) with the following topology: (*Hemipilia gracilis* + *Hemipilia yajiangensis*; PP 1.00, BP_ML_ 100) + (*Vietorchis aurea* + *Vietorchis furcata*; PP 1.00, BP_ML_ 100).

### Plastome of *Vietorchis furcata*: structure, gene content and selective pressure

**Figure 6:**
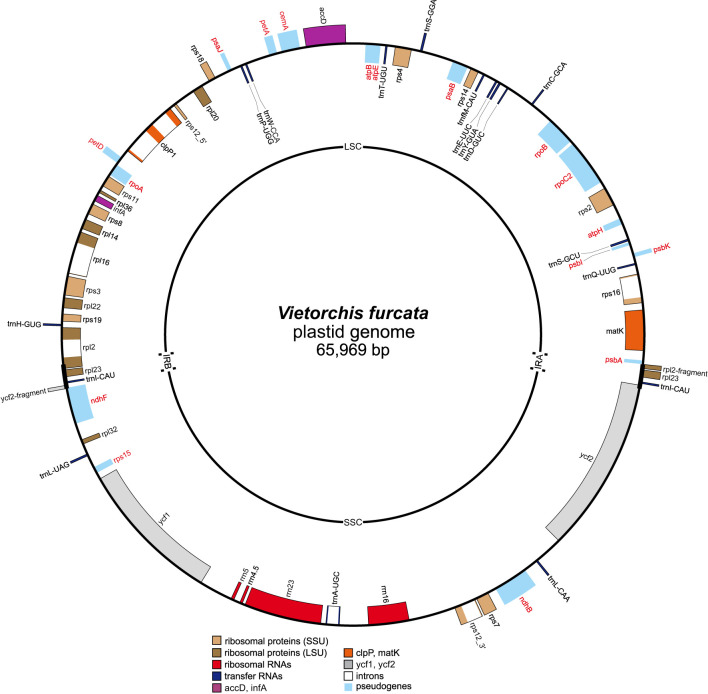
Circular map of the plastid genome of *Vietorchis furcata*. Genes shown outside of the circle are transcribed counterclockwise and those inside are transcribed clockwise. Names of pseudogenes are marked with red color. LSC, large single copy region; SSC, small single copy region; IRA and IRB, inverted repeats A and B, respectively.

The newly assembled complete plastid genome of *Vietorchis furcata* is 65,969 base pairs (bp) in length with the typical quadripartite structure containing a large (LSC) and a small single copy (SSC) region (35,123 and 29,056 bp, respectively) separated with inverted repeats (IR) of 895 bp (**Figure 6**). The overall GC content was 33.58%.

Forty five unique genes were revealed in the plastome (**Table 3**), including those of 4 rRNAs, 16 tRNAs, 11 proteins of small ribosomal subunit, 8 proteins of large ribosomal subunit, and 6 other proteins. Of these genes, 31 were situated in the LSC, 11 were in the SSC, and the trans-spliced *rps12* was spread in both single copy regions. The inverted repeats contained two genes (*rpl23* and *trnI-CAU*) and partial sequences of *rpl2* and *ycf2* genes (with their other parts being in the LSC and SSC, respectively). Fraction of the coding DNA was 44.01%.

**Table 3:** Gene content in the plastome of *Vietorchis furcata*.

Gene groups	Gene names
NDH complex	*-*
Photosynthesis	*-*
ATP synthesis	*-*
Plastid RNA polymerase subunits	*-*
Ribosomal RNA genes	*rrn5, rrn4.5, rrn16, rrn23*
Transfer RNA genes	trnA-UGC, trnC-GCA, trnD-GUC, trnE-UUC, trnfM-CAU, trnH-GUG, trnI-CAU^*^, trnL-CAA, trnL-UAG, trnP-UGG, trnQ-UUG, trnS-GCU, trnS-GGA, trnT-UGU, trnW-CCA, trnY-GUA
Small subunit ribosomal proteins	*rps2, rps3, rps4, rps7, rps8, rps11, rps12, rps14, rps16, rps18, rps19*
Large subunit ribosomal proteins	rpl2, rpl14, rpl16, rpl20, rpl22, rpl23^*^, rpl32, rpl36
Maturase	*matK*
Subunit of acetyl-CoA carboxylase	*accD*
ATP-dependent protease, subunit P	*clpP*
Translational initiation factor	*infA*
Conserved open reading frames (*ycf*)	*ycf1, ycf2*

The genes located in inverted repeats are marked with asterisks.

No intact photosynthesis-related genes were found, but 17 pseudogenes were annotated in the plastome: *atpB*, *atpE*, *atpH*, *cemA*, *rpoA*, *rpoB*, *rpoC2*, *ndhB*, *ndhF*, *petA*, *petD*, *psaB*, *psaJ*, *psbA*, *psbI*, *psbK*, and *rps15* (**Figure 6**); all of them except for *rps15* are derived from the photosynthesis-related genes. All of the pseudogenes contained multiple internal termination codons. Of the six intron-containing genes, five were protein-coding sequences (*rps16*, *rpl16*, *rpl2*, *rps12*, *clpP*; the last one contained two introns) and one was the tRNA gene (*trnA-UGC*).

The genes and pseudogenes in the plastome of *V. furcata* retained the same relative position as in the phylogenetically closest known plastomes, i.e. those of the photosynthetic *Hemipilia gracilis* and *Hemipilia yajiangensis* (**Supplementary Figure 13**).

A total of 13 dispersed repeats were found in the plastome of *V. furcata*, with the longest repeat being 68 bases long (**Supplementary Figure 14**). The numbers of direct, inverted and palindrome repeats were almost equal: 4, 4 and 5, respectively. Comparison of the plastomes of *Vietorchis furcata* and *Hemipilia yajiangensis* showed similar relative amount of dispersed repeats (~0.20 vs. ~0.19 repeats per 1000 bases) and their relative length (0.50% vs. 0.47% of the plastome length).

The likelihood ratio tests (LRTs) based on the branch models (and performed using CodeML) showed that the “single ω model” was preferable for 23 (of 25) protein-coding genes, whereas the difference in ω values between the foreground and background branches can be considered statistically significant (implying the alternative model) for the two remaining genes (*rps2* and *rps11*, **Table 4**). However, for all the genes, an averaged omega ratio was below 1, suggesting that all of them undergo negative selection. The performed branch-site tests also did not reveal any signs of positive selection acting on the amino acid residues (**Supplementary Table 1**). Similarly, the LRTs performed using BUSTED showed no evidence of episodic diversifying selection in any of the analyzed genes in the plastome of *V. furcata* (**Table 4**).

**Table 4:** Nonsynonymous (Dn) to synonymous (Ds) substitution rate ratio for plastid gene sequences in the photosynthetic lineages of the subfamily Orchidoideae studied here and in *Vietorchis furcata*.

Gene names	Dn/Ds ratio in photosynthetic lineages (PAML)	Dn/Ds ratio in *Vietorchis furcata* (PAML)	LRT *p*-value (PAML)	LRT *p*-value (BUSTED)
*accD*	0.4661	0.3156	0.1492	0.50
*clpP*	0.0884	0.1678	0.2820	0.50
*infA*	0.0573	0.1788	0.1716	0.50
*matK*	0.4562	0.6255	0.2042	0.47
*rpl14*	0.1144	0.4381	0.0459	0.50
*rpl16*	0.1938	0.2353	0.7362	0.50
*rpl2*	0.2622	0.2108	0.7211	0.50
*rpl20*	0.3854	0.5069	0.5860	0.28
*rpl22*	0.2649	0.508	0.2601	0.50
*rpl23*	0.1322	0.8199	0.0368	0.50
*rpl32*	0.2493	0.5702	0.3551	0.32
*rpl36*	0.1594	0.3429	0.4964	0.50
*rps11*	0.1108	0.3899	**0.0103	0.50
*rps12*	0.1626	0.5786	0.1714	0.50
*rps14*	0.2570	0.2526	0.9789	0.50
*rps16*	0.1719	0.2997	0.3906	0.50
*rps18*	0.1462	0.5851	0.0312	0.50
*rps19*	0.2742	0.1985	0.7472	0.50
*rps2*	0.1115	0.4894	**0.0013	0.26
*rps3*	0.1470	0.3548	0.0347	0.19
*rps4*	0.2017	0.5588	0.0271	0.50
*rps7*	0.3321	0.5061	0.6065	0.50
*rps8*	0.1525	0.3366	0.1044	0.42
*ycf1*	0.5984	0.5630	0.6778	0.50
*ycf2*	0.7460	0.6457	0.3707	0.50

** indicates that the differеnce is statistically significant after Bonferroni correction.

## Discussion

### Subfamily placement of *Vietorchis*, and homoplastic evolution of rhizomes in mycoheterotrophic Orchidaceae

In our reconstructions, *Vietorchis* is deeply nested within the subfamily Orchidoideae, and thus the earlier discussed possibility of the placement of *Vietorchis* outside Orchidoideae is now decisively refuted. Taking into account the implied close relationship between *Vietorchis* and *Silvorchis*, we extrapolate this conclusion to the latter genus. The reason of doubts regarding the subfamily placement of these two genera was the structure of their underground organs, i.e. the “*Epipogium*-*Cyrtosia*-like” plagiotropic thick fleshy branching rootless tuber-like rhizomes (in *S. colorata* and in the genus *Vietorchis*; Averyanov et al., 2013) or “*Gastrodia*-like” rhizome-like tubers (in *S. vietnamica*; Averyanov et al., 2018). It is noteworthy that Smith (1907) described the underground organs of *S. colorata* as short fleshy rhizome bearing 0.6 cm thick roots. This was reproduced in subsequent accounts (e.g. Pridgeon et al., 2005) since no material was available to clarify the morphology. Similarly, Averyanov (2010) initially described *V. aurea* as having tuber-like roots due to the lack of appropriate material at that time. Based on the newly obtained material of *Silvorchis* and *Vietorchis*, as well as on the drawing from the protologue of *S. colorata* (Smith, 1907), Averyanov et al. (2013, 2018) concluded that the two genera share fleshy rootless underground stems ranging from thick rhizomes to somewhat elongate tubers. There is also a possibility that the underground plant part in *Silvorchis* and *Vietorchis* is a root-stem tuberoid (see also Averyanov et al., 2023), i.e. a storage root with its basal portion surrounding a core of stem tissue with a bud (as defined by Dressler, 1993), but anyway these genera lack morphologically distinct roots of typical shape and structure.

Thus, our molecular phylogenetic data support the idea that the similarities in the rhizome morphology between *Vietorchis* (and the supposedly related *Silvorchis*) and the genera from the subfamilies Epidendroideae and Vanilloideae are examples of a convergence, and are likely caused by the mycoheterotrophic lifestyle shared by these genera. At the same time, the rhizomes of *Vietorchis* are markedly different from those of its closest photosynthetic relatives within Orchidoideae. In particular, *Sirindhornia*, the closest genus to *Vietorchis* in our reconstructions based on the datasets employing selected nuclear and plastid regions, is characterized by root-bearing tubers/tuberoids (Pedersen et al., 2003; Chen et al., 2009; Pedersen, 2011).

### Relationships of *Vietorchis* within the subtribe Orchidinae

Our reconstructions based on the nuclear dataset and two plastid datasets demonstrate that *Vietorchis* is placed within the subtribe Orchidinae, where it (together with *Sirindhornia*, when included in analysis) forms a sister group to *Hemipilia* s.l. (including *Ponerorchis* and *Tsaiorchis*). Molecular phylogenetic evidence therefore supports the synonymization of the subtribe Vietorchidinae with Orchidinae suggested by Olędrzyńska et al. (2016). Since the latter authors have not indicated the reasons of their decision, our study is the first one to provide a basis for such a taxonomic rearrangement.

In the two-marker trees (i.e. those based on ITS+*Xdh* and *matK*+*psb*A-*trn*H datasets), which employed a broader sampling with respect to the trees based on the plastomes, *Vietorchis* is most close to *Sirindhornia*. *Vietorchis* is therefore inferred here to be a part of the clade XVIII recognized by Jin et al. (2017). All the three species of *Sirindhornia* are restricted to limestone mountains (Pedersen et al., 2003; Pedersen, 2011), and one species of *Vietorchis* (*V. aurea*) also inhabits limestone karsts. With this evidence, our phylogenetic results suggest that association with limestone is possibly a plesiomorphic condition for *Vietorchis*. This assumption is to be tested after establishment of the phylogenetic position of *Silvorchis*, a putative closest relative of *Vietorchis* known exclusively in non-limestone areas.

Morphologically, *Vietorchis* is unique among Orchidinae (and among the entire tribe Orchideae) in having a lip with a massive callus and a raised longitudinal keel (Averyanov, 2013; Averyanov et al., 2013). In addition, *Vietorchis* and *Silvorchis* are remarkable in their spurless lips, which is a very rare feature in Orchidinae (Dressler, 1993), known, for example, in *Ophrys* and *Serapias* L. No morphological proximity between *Vietorchis* and *Sirindhornia* has ever been proposed. Indeed, with respect to the flower structure, *Vietorchis* (together with *Silvorchis*, for which the molecular phylogenetic data are still lacking) is equally close to a number of genera traditionally associated with *Orchis* as Orchidinae s.s., of which the ones inhabiting tropical mainland Asia being *Sirindhornia* and *Hemipilia* s.l. (including *Ponerorchis*, *Tsaiorchis*, the formerly recognized *Amitostigma* Schltr. and *Neottianthe* (Rchb.) Schltr. and possibly *Apetalanthe* Aver. & Vuong, the latter genus not yet included in a phylogenetic analysis). All genera of this alliance share such characters as small plant habit, fleshy tuber-like roots, erect anther with closely spaced almost parallel thecae, thecae with short bases supported by small rostellum having no rostellar arms, large hemispheric or conoid auricles on the sides of the anther, clavate pollinia with long caudicles terminated by closely spaced viscidia, viscidia completely separated or united into a single body, viscidia naked or covered by bursiculum (or bursicula), entire concave stigma without any extensions. Except for the roots, this set of characters is also found in *Vietorchis* and *Silvorchis*.

Thus, flower morphology is concordant with the molecular data in placing *Vietorchis* into Orchidinae, but appears to be only moderately instructive in determination of its phylogenetic relationships within the subtribe. Morphology strongly supports the assignment of *Vietorchis* to the second major clade (the one containing *Orchis*, etc), but fails to guide its affinities within the clade. At the same time, the two subclades of this clade are correlated with the geographical evidence, and *Vietorchis* fits this pattern as it occupies the predominantly tropical subclade. Therefore, characteristics of flower morphology combined with the geographical distribution exhibit considerable phylogenetic signal.

### Plastid genome of *Vietorchis furcata* in comparison with other reduced plastomes

While the typical plastomes of autotrophic angiosperms are 120–170 kb in length and encode 120–130 genes (Ruhlman and Jansen, 2021), the plastome of the non-photosynthetic *Vietorchis furcata* is substantially reduced in both length and gene content, in line with the tendency observed in heterotrophic plants (Barrett and Davis, 2012; Barrett et al., 2014; Wicke et al., 2016; Graham et al., 2017). *Vietorchis furcata* possesses one of the most reduced plastomes in the subfamily Orchidoideae (65969 bp), the other ones being those of the two other fully mycoheterotrophic species, *Rhizanthella gardneri* R.S.Rogers (59190 bp: Delannoy et al., 2011) and *Corybas cryptanthus* Hatch (69300 bp: Murray, 2019). The plastome of *Vietorchis furcata* lacks intact photosynthesis-related genes and contains some genes of ribosome components and transfer RNAs in addition to several other “housekeeping” genes (*matK*, *accD*, *clpP*, *infA*, *ycf1*, *ycf2*). The retained protein-coding genes in *Vietorchis furcata* appear to undergo the same negative selection as in its photosynthetic relatives from the subfamily Orchidoideae, although the stabilizing constraints are likely to be relaxed in the *rps2* and *rps11* sequences in *V. furcata*.

In terms of functional gene content, the plastome of *Vietorchis furcata* seems to be at one of the last stages of degradation (sensu Barrett et al., 2014), because it has lost several genes of tRNA and the *rps15* ribosomal protein has been pseudogenized. Similar gene content is characteristic of the most reduced plastomes in Orchidaceae, e.g., in *Epipogium* (Schelkunov et al., 2015), *Gastrodia* (Wen et al., 2022), *Pogoniopsis* (Klimpert et al., 2022), *Rhizanthella* (Delannoy et al., 2011). However, in spite of their similar gene content, the plastomes of *Epipogium*, *Gastrodia* and *Pogoniopsis* are considerably shorter than those of *Rhizanthella* and *Vietorchis*. This is due to a larger fraction of non-coding DNA in the plastomes of *Rhizanthella* and *Vietorchis*, and particularly, the presence of multiple pseudogenes in the latter. Pseudogenes are usually rapidly purged from the plastomes of heterotrophic plants, and their abundant presence is associated with very recent transition to heterotrophy (see e.g. Barrett et al., 2014; Samigullin et al., 2016). The other types of non-coding DNA (intergenic spacers and introns) are also scarce in highly reduced plastomes (Schelkunov et al., 2015, 2019; Su et al., 2019). Thus, the plastome of *Vietorchis* represents an interesting example of heterochrony: it has a highly reduced gene set (with the reduction affecting even the ribosomal protein genes, which is typical for the last steps of plastome degradation, following the model of Barrett and Davis, 2012) but keeps a large amount of pseudogenes and non-coding DNA of the other types (which is typical for the earliest steps of degradation). It should also be noted that though ribosome is a necessary component of virtually any cell (and plastids and mitochondria as well, since they are derivatives of a bacterial cell), the essentiality of different ribosomal proteins is considerably unequal (see Nikolaeva et al., 2021). It might be beneficial to update the model of plastome reduction in heterotrophic plants taking this idea into account.

In the phylogenetic trees based on the plastid datasets, both studied species of *Vietorchis* occupy long terminal branches, which assume a substantial overall elevation of substitution rates. The phenomenon of long branches is characteristic of plastomes of many heterotrophic lineages (Lam et al., 2018), and the elevated substitution rates in plastomes of fully heterotrophic plants seem to be frequent (see a review by Sanchez-Puerta et al., 2023), although not obligatory (e.g., *Cephalanthera humilis* X.H.Jin: Lam et al., 2018, *Petrosavia stellaris* Becc.: Logacheva et al., 2014).

The small length of the inverted repeats in *Vietorchis furcata* (895 bp) is a remarkable feature, which is shared by this species with *Epipogium roseum* Lindl. (about 250–300 bp in different accessions: Schelkunov et al., 2015) and *Pogoniopsis schenckii* Cogn. (1509 bp: Klimpert et al., 2022). The IR reduction is the only apparent structural alteration in the plastome of *Vietorchis furcata* with respect to the typical plastomes of photosynthetic angiosperms. Both large contractions and expansions of the IRs are often documented in heterotrophic plant lineages (Wicke et al., 2013; Feng et al., 2016; Logacheva et al., 2016; Kim et al., 2020b; Yudina et al., 2021), sometimes even within a single genus (e.g., *Epipogium*: Schelkunov et al., 2015, *Neottia* Guett.: Feng et al., 2016, *Thismia* Griff.: Yudina et al., 2021).

The retention of plastome gene order revealed in *Vietorchis furcata* is rather typical for the species with recent transition to heterotrophy and low degree of plastome reduction (e.g. Barrett et al., 2014; Samigullin et al., 2016). And vice versa, numerous deeply reduced plastomes of mycoheterotrophic (e.g., Schelkunov et al., 2015; Lim et al., 2016; Li et al., 2019; Yudina et al., 2021; Wen et al., 2022) as well as holoparasitic (e.g., Schelkunov et al., 2019; Su et al., 2019) species show a highly altered gene order, although the retained colinearity was also demonstrated for some of such taxa (e.g., Logacheva et al., 2011; Lam et al., 2015; Klimpert et al., 2022). Thus, the plastome of *Vietorchis furcata*, being colinear to the plastomes of the autotrophic genus *Hemipilia* (which are the most close phylogenetically to *Vietorchis* among the available orchid plastomes) again demonstrates the same heterochronic pattern as outlined above. It should be noted, however, that the major determinant of the rearrangements (inversions and translocations) in plant plastomes are the dispersed repeats, and therefore the number of such rearrangements relates largely to the repeat richness, and not to the nutrition type. Indeed, numerous autotrophic plant groups with highly rearranged plastomes are known (e.g. within Campanulaceae: Haberle et al., 2008, Ericaceae: Logacheva et al., 2016, Geraniaceae: Guisinger et al., 2011, Oleaceae: Lee et al., 2007); all of these plastomes have high fraction of repetitive DNA, which is supposed in the cited studies to relieve rearrangements. The presence of abundant dispersed repeats seems to be one of the necessary conditions for the occurrence of rearrangements; in line with this idea, the unrearranged plastome of *Vietorchis furcata* has low abundance of repeats.

## Conclusions

We provided for the first time the results of molecular phylogenetic analysis of *Vietorchis*, a genus of mycoheterotrophic orchids with unusual morphology and continuously debated evolutionary relationships. The obtained results were largely obtained through the high-throughput sequencing approaches and covered two of the three species of the genus. We confirmed that *Vietorchis* is a member of the subfamily Orchidoideae, which implies a homoplastic evolution of orchid subterranean shoots related to transitions to heterotrophy: the similarities of the rhizomes of *Vietorchis* to those of the mycoheterotrophic taxa of Epidendroideae and Vanilloideae are proved to have a convergent nature.

Our study demonstrated that *Vietorchis* belongs to the tribe Orchideae, where it is deeply nested within the subtribe Orchidinae. Our findings therefore corroborate the necessity of synonymization of the subtribe Vietorchidinae with Orchidinae. The inclusion of *Vietorchis* into one of the two major clades of Orchidinae is in strong agreement with floral morphology. *Vietorchis* is shown to be phylogenetically placed in the vicinity of the species-rich genus *Hemipilia* and sister to *Sirindhornia* (both genera being entirely autotrophic). Among the members (and putative members) of the subfamily Orchidoideae not included into the phylogenetic analysis, the poorly known mycoheterotrophic genus *Silvorchis* is the only one that has ever been proposed to be allied to *Vietorchis*. The currently available morphological evidence suggest a sister relationship between *Silvorchis* and *Vietorchis* (and then these two genera are sister to *Sirindhornia*); however, the phylogenetic placement of *Silvorchis* is to be verified by utilizing molecular data, along with the question of a common versus independent transition to heterotrophy in *Silvorchis* and *Vietorchis*.

Finally, we characterized the plastid genome of one of the species of *Vietorchis*, *V. furcata*, and performed its comparative analysis with plastomes of other mycoheterotropic as well as autrotrophic orchids. The plastome is found to be 65969 bp long and comprise 45 unique genes along with 17 pseudogenes. On the one side, the plastome structure is typical for a non-photosynthetic plant in a lack of any functional photosynthesis-related genes. On the other side, however, the plastome structure demonstrates unusual heterochronic patterns expressed in co-occurring of a highly reduced gene set with the retention of pseudogenes and other non-coding DNA and the absence of rearrangements compared with the closest studied autotrophic species.

## Data availability statement

The datasets presented in this study can be found in online repositories. The names of the repository/repositories and accession number(s) can be found below: Nucleotide sequences generated in this study are available in the GenBank online public database (https://www.ncbi.nlm.nih.gov/genbank/) under the accession numbers OQ318186–OQ318196, OQ318871, OQ331227, OQ331229, OQ331230, OQ344206, OQ344207, OQ344208, OQ352447.

## Author contributions

TS: Data curation, Formal analysis, Investigation, Visualization, Writing – original draft. ML: Conceptualization, Data curation, Formal analysis, Investigation, Project administration, Supervision, Writing – original draft. LA: Investigation, Resources, Writing – review & editing. S-JZ: Investigation, Resources, Writing – review & editing. L-FF: Investigation, Writing – review & editing. MN: Conceptualization, Funding acquisition, Investigation, Project administration, Resources, Supervision, Writing – original draft.

## Appendix A1

**APPENDIX TABLE 1:** Nuclear and plastid regions used in this study: species sampled, voucher information, geographical origin, and GenBank (NCBI) accession numbers. Asterisk denotes newly generated sequences.

Species	Voucher	Source or label data	GenBank number for ITS	GenBank number for *Xdh*	GenBank number for *matK*	GenBank number for *psb*A*-trn*H (including *rps*19)
*Anacamptis* sp.	*1351*	ITS, *Xdh*, *matK*, *psb*A*-trn*H: Jin et al. (2017)	MF944260	MF945073	MF945394	MF944716
*Brachycorythis henryi* (Schltr.) Summerh.	*X.H. Jin 13860* (PE)	ITS, *Xdh*, *matK*, *psb*A*-trn*H: Jin et al. (2017)	MF944262	MF945034	MF945438	MF944675
*Brachycorythis obcordata* (Lindl. ex Wall.) Summerh.	*B.B. Raskoti 2014106* (KATH)	ITS, *Xdh*, *matK*, *psb*A*-trn*H: Jin et al. (2017)	MF944263	MF945098	MF945500	MF944742
*Dactylorhiza maculata* ssp. *fuchsii* (Druce) Hyl.	*X.H. Jin 14625* (PE)	ITS, *Xdh*, *matK*, *psb*A*-trn*H: Jin et al. (2017)	MF944265	MF944992	MF945400	MF944633
*Dactylorhiza viridis* (L.) R.M.Bateman, Pridgeon & M.W.Chase	China, Yunnan, *STET 0772* (PE)	ITS: Jin et al. (2012); *Xdh*, *psb*A*-trn*H: Jin et al. (2017); *matK*: Jin et al. (2014)	JN696446	MF945117	KJ452797	MF944762
*Dactylorhiza* sp.	*1245 / X.H. Jin 14263* (PE)	ITS, *Xdh*, *matK*, *psb*A*-trn*H: Jin et al. (2017)	MF944266	MF944993	MF945401	MF944634
*Diplomeris pulchella* D.Don	*X.H. Jin, L. Zhang 11553* (PE)	ITS, *Xdh*, *matK*, *psb*A*-trn*H: Jin et al. (2017)	MF944270	MF945181	MF945535	MF944826
*Galearis spathulata* (Lindl.) P.F.Hunt	*X.H. Jin, W.T. Jin, S.Z. Xu 13234* (PE)	ITS, *matK*: Jin et al. (2014); *Xdh*, *psb*A*-trn*H: Jin et al. (2017)	KJ460094	MF945156	KJ452850	MF944801
*Galearis tschiliensis* (Schltr.) P.J.Cribb, S.W.Gale & R.M.Bateman	*STET 09281* (PE)	ITS, *matK*: Jin et al. (2014); *Xdh*, *psb*A*-trn*H: Jin et al. (2017)	KJ460057	MF945128	KJ452813	MF944773
*Galearis wardii* (W.W.Sm.) P.F.Hunt	*X.H. Jin, W.T. Jin, Y.Q. Cui 14576* (PE)	ITS, *Xdh*, *matK*, *psb*A*-trn*H: Jin et al. (2017)	MF944274	MF945011	MF945417	MF944652
*Gennaria griffithii* (Hook.f.) X.H.Jin & D.Z.Li	China, Yunnan, *X.H. Jin 10879* (PE)	ITS, *matK*: Jin et al. (2012); *Xdh*, *psb*A*-trn*H: Jin et al. (2017)	JN696445	MF944987	JN696430	MF944627
*Gymnadenia conopsea* (L.) R.Br.	China, Yunnan, *X.H. Jin 9896* (PE)	ITS: Jin et al. (2012); *Xdh*, *psb*A*-trn*H: Jin et al. (2017); *matK*: Jin et al. (2014)	JN696449	MF945115	KJ452795	MF944760
*Gymnadenia orchidis* Lindl.	*X.H. Jin, W.T. Jin, Y.Q. Cui 14459* (PE)	ITS, *Xdh*, *matK*, *psb*A*-trn*H: Jin et al. (2017)	MF944276	MF945088	MF945491	MF944732
*Habenaria aitchisonii* Rchb.f.	*X.H. Jin, W.T. Jin, Y.Q. Cui 14680* (PE)	ITS, *Xdh*, *matK*, *psb*A*-trn*H: Jin et al. (2017)	MF944279	MF945017	MF945422	MF944658
*Habenaria delavayi* Finet	*X.H. Jin 10396* (PE)	ITS, *Xdh*, *matK*, *psb*A*-trn*H: Jin et al. (2017)	MF944290	MF945056	MF945461	MF944699
*Habenaria fordii* Rolfe	*W.T. Jin, Y.Q. Cui* 14362 (PE)	ITS, *Xdh*, *matK*, *psb*A*-trn*H: Jin et al. (2017)	MF944294	MF945027	MF945431	MF944668
*Habenaria intermedia* D.Don	*B.B. Raskoti 201446* (KATH)	ITS, *Xdh*, *matK*, *psb*A*-trn*H: Jin et al. (2017)	MF944298	MF945094	MF945496	MF944738
*Habenaria marginata* Colebr.	*B.B. Raskoti 201459* (KATH)	ITS, *Xdh*, *matK*, *psb*A*-trn*H: Jin et al. (2017)	MF944307	MF945101	MF945503	MF944745
*Habenaria pantlingiana* Kraenzl.	*B.B. Raskoti 201335* (KATH)	ITS, *Xdh*, *matK*, *psb*A*-trn*H: Jin et al. (2017)	MF944309	MF945118	MF945513	MF944763
*Habenaria pectinata* D.Don	*X.F. Gao, Z.M. Zhu, W.B. Ju, W.T. Jin 14714* (PE)	ITS, *Xdh*, *matK*, *psb*A*-trn*H: Jin et al. (2017)	MF944310	MF945185	MF945539	MF944830
*Habenaria tibetica* Schltr.	*X.H. Jin, W.T. Jin, S.Z. Xu 13108* (PE)	ITS, *Xdh*, *matK*, *psb*A*-trn*H: Jin et al. (2017)	MF944325	MF945177	MF945531	MF944822
*Hemipilia calophylla* C.S.P.Parish & Rchb.f.	*W.T. Jin 11798* (PE)	ITS, *matK*: Jin et al. (2014); *Xdh*, *psb*A*-trn*H: Jin et al. (2017)	KJ460095	MF945158	KJ452852	MF944803
*Hemipilia chusua* (D.Don) Y.Tang & H.Peng 1	*X.H. Jin 8272* (PE)	ITS, *Xdh*, *matK*, *psb*A*-trn*H: Jin et al. (2017)	MF944401	MF945055	MF945460	MF944698
*Hemipilia chusua* (D.Don) Y.Tang & H.Peng 2	*X.H. Jin 9138* (PE)	ITS, *Xdh*, *matK*, *psb*A*-trn*H: Jin et al. (2017) (listed as *Ponerorchis nana* (King & Pantl.) Soó)	MF944404	MF945070	MF945475	MF944713
*Hemipilia cordifolia* Lindl.	*X.H. Jin 11052* (PE)	ITS, *Xdh*, *matK*, *psb*A*-trn*H: Jin et al. (2017) (listed as *Hemipilia cruciata* Finet)	MF944330	MF945057	MF945462	MF944700
*Hemipilia gracilis* (Blume) Y.Tang, H.Peng & T.Yukawa	China, Yunnan, *X.H. Jin 9257* (PE)	ITS: Jin et al. (2014); *Xdh*, *psb*A*-trn*H: Jin et al. (2017); *matK*: Jin et al. (2012)	KJ460036	MF945003	JN696435	MF944644
*Hemipilia pinguicula* (Rchb.f. & S.Moore) Y.Tang & H.Peng	*W.T. Jin 16006* (PE)	ITS, *Xdh*, *matK*, *psb*A*-trn*H: Jin et al. (2017)	MF944417	MF945093	MF945495	MF944737
*Hemipilia purpureopunctata* (K.Y.Lang) X.H.Jin, Schuit. & W.T.Jin	*X.H. Jin 13198* (PE)	ITS, *matK*: Jin et al. (2014); *Xdh*, *psb*A*-trn*H: Jin et al. (2017)	KJ460051	MF945123	KJ452807	MF944768
*Hemipilia tibetica* (Schltr.) Y.Tang & H.Peng	*X.H. Jin, L. Zhang 11075* (PE)	ITS, *Xdh*, *matK*, *psb*A*-trn*H: Jin et al. (2017)	MF944412	MF945043	MF945449	MF944685
*Herminium coiloglossum* Schltr.	*X.H. Jin 8273* (PE)	ITS, *matK*, *psb*A*-trn*H: Raskoti al. (2016); *Xdh*: Jin et al. (2017)	KR350153	MF945161	KR350189	KR350299
*Herminium fallax* (Lindl.) Hook.f.	*X.H. Jin, W.T. Jin, S.Z. Xu 13248* (PE)	ITS, *Xdh*, *matK*, *psb*A*-trn*H: Raskoti al. (2016)	KR350164	KR350453	KR350200	KR350310
*Herminium forceps* (Finet) Schltr.	*X.H. Jin 9360* (PE)	ITS: Jin et al. (2012); *Xdh*, *psb*A*-trn*H: Raskoti al. (2016); *matK*: Jin et al. (2014)	JN696461	KR350423	KJ452789	KR350280
*Herminium tibeticum* X.H.Jin, Schuit. & Raskoti	China, Tibet, *STET 1293* (PE)	ITS, *matK*: Jin et al. (2012) (listed as *Herminium monorchis* (L.) R.Br.); *Xdh*, *psb*A*-trn*H: Raskoti al. (2016) (listed as *Herminium* sp.)	JN696453	KR350417	JN696439	KR350273
*Hsenhsua chrysea* (W.W.Sm.) X.H.Jin, Schuit., W.T.Jin & L.Q.Huang	*X.H. Jin, L. Zhang 11082* (PE)	ITS, *matK*: Jin et al. (2014); *Xdh*, *psb*A*-trn*H: Raskoti al. (2016)	KJ460056	KR350430	KJ452812	KR350286
*Ophrys apifera* Huds.	ITS, *matK*: *M.W. Chase 536* (K); *psb*A*-trn*H: England, *M.W. Chase 13839* (K)	ITS, *matK*: Salazar et al. (2003); *psb*A*-trn*H: Devey et al. (2008)	AJ539529	–	AJ543953	AM711642
*Ophrys insectifera* L.	unknown	ITS, *Xdh*, *matK*, *psb*A*-trn*H: Jin et al. (2017)	MF944348	MF945090	MF945396	MF944734
*Orchis militaris* L.	Switzerland, Valais, *A. Widmer* (Z/ZT)	ITS: Soliva et al. (2001); *matK*: unpublished	AY014548	–	KF997352	–
*Orchis simia* Lam.	(NAP)	ITS: Aceto et al. (1999); *matK*: unpublished	Z94107+ Z94108	–	KF997476	–
*Pecteilis susannae* (L.) Raf.	*X.H. Jin, W.T. Jin, Y.Q. Cui 14631* (PE)	ITS, *Xdh*, *matK*, *psb*A*-trn*H: Jin et al. (2017)	MF944350	MF945018	MF945423	MF944659
*Peristylus affinis* (D.Don) Seidenf.	*X.H. Jin, W.T. Jin, Y.Q. Cui 14623* (PE)	ITS, *Xdh*, *matK*, *psb*A*-trn*H: Jin et al. (2017)	MF944353	MF944999	MF945406	MF944640
*Peristylus lacertifer* (Lindl.) J.J.Sm.	*X.H. Jin 11645* (PE)	ITS, *Xdh*, *matK*, *psb*A*-trn*H: Jin et al. (2017)	MF944365	MF945074	MF945477	MF944717
*Platanthera brevicalcarata* Hayata	*X.H. Jin 10024* (PE)	ITS, *matK*: Jin et al. (2014); *Xdh*, *psb*A*-trn*H: Jin et al. (2017)	KJ460067	MF945136	KJ452823	MF944781
*Platanthera fuscescens* (L.) Kraenzl.	China, Yunnan, *X.H. Jin 10385* (PE)	ITS: Jin et al. (2012); *Xdh*, *psb*A*-trn*H: Jin et al. (2017); *matK*: Jin et al. (2014)	JN696467	MF945109	KJ452788	MF944754
*Platanthera sparsiflora* (S.Watson) Schltr.	*B. Bartholomew, A. Anderson, H.W. Li, T.S. Ying 2313* (PE)	ITS, *Xdh*, *matK*, *psb*A*-trn*H: Jin et al. (2017)	MF944389	MF945102	MF945504	MF944746
*Platanthera taiwanensis* (S.S.Ying) S.C.Chen, S.W.Gale & P.J.Cribb	*Hsu 5942* (TAIF)	ITS, *Xdh*, *matK*, *psb*A*-trn*H: Jin et al. (2017)	MF944393	MF945038	MF945443	MF944680
*Ponerorchis limprichtii* (Schltr.) Soó	*X.H. Jin, W.T. Jin, Y.Q. Cui 14466* (PE)	ITS, *Xdh*, *matK*, *psb*A*-trn*H: Jin et al. (2017)	MF944398	MF945020	MF945425	MF944661
*Satyrium yunnanense* Rolfe	*X.H. Jin, W.T. Jin, Y.Q. Cui 14710* (PE)	ITS, *Xdh*, *matK*, *psb*A*-trn*H: Jin et al. (2017)	MF944415	MF945086	MF945489	MF944730
*Sirindhornia monophylla* (Collett & Hemsl.) H.A.Pedersen & Suksathan	China, Yunnan, *Z.J. Liu 7951* (NOCC)	Dehong Dai and Jingpo Autonomous Prefecture, Mangshi City, 26.05.2014	OQ331227*	OQ344206*	OQ344208*	–
*Sirindhornia pulchella* H.A.Pedersen & Indham.	*X.H. Jin 11012* (PE)	ITS, *matK*: Jin et al. (2014); *Xdh*, *psb*A*-trn*H: Jin et al. (2017)	KJ460045	MF945119	KJ452801	MF944764
*Tsaiorchis keiskeoides* (Gagnep.) X.H.Jin, Schuit. & W.T.Jin	China, Yunnan, *YNET 507* (PE)	ITS: Jin et al. (2012); *Xdh*, *psb*A*-trn*H: Jin et al. (2017); *matK*: Jin et al. (2014)	JN696466	MF945114	KJ452794	MF944759
*Vietorchis aurea* Aver. & Averyanova	Vietnam, Ninh Binh, *May Van Xinh MVX 261* (LE: LE01076723)	Cuc Phuong National Park, 200‒300 m, 05.2004	OQ331229*	–	OQ318186*	OQ352447*
*Vietorchis furcata* Aver. & Nuraliev	Vietnam, Dak Lak, *M.S. Nuraliev 998* (LE: LE01076759)	Lak distr., Bong Krang munic., Chu Yang Sin national park, 12 km S of Krong Kmar village, forest, near small river, c. 1100 m, 12°23’41”N 108°20’55”E, 28.05.2014	OQ331230*	OQ344207*	OQ318871*	OQ318871*
**Outgroups**						
*Disperis* sp.	*1520 / CPG 29216* (PE)	ITS, *Xdh*, *matK*, *psb*A*-trn*H: Jin et al. (2017)	MF944272	MF945082	MF945484	MF944725
*Goodyera schlechtendaliana* Rchb.f.	South Korea, Jeju, *N. Lee 0510011* (EWH)	ITS, *psb*A*-trn*H: Lee et al. (2012); *matK*: unpublished	HM021571	–	KC704635	HM021618
*Spiranthes sinensis* (Pers.) Ames	eastern Asia, *M.W. Chase 10450* (K)	ITS, *matK*: Salazar and Jost (2012); *Xdh*: unpublished	HE575518	–	HE575508	KC704395

Asterisk denotes newly generated sequences.

## Appendix A2

**APPENDIX TABLE 2:** Plastid genomes used in this study: species sampled, voucher information, geographical origin, and GenBank (NCBI) accession numbers. In cases where two numbers are indicated, the first one is the accession containing a reference to the original plastome annotation. Asterisk denotes newly generated sequences.

Species	Voucher	Source or label data	GenBank reference for plastid genome
*Dactylorhiza majalis* (Rchb.) P.F.Hunt & Summerh.	Poland, *SG-13237* (UGDA)	May et al. (2019)	NC_044644 MK984209
*Dactylorhiza viridis* (L.) R.M.Bateman, Pridgeon & M.W.Chase	unknown	unpublished	NC_056192 MN824430
*Galearis cyclochila* (Franch. & Sav.) Soó	*PDBK2000-0786* (KB, KUS)	Kim et al. (2020b)	NC_046818 MN200388
*Gymnadenia conopsea* (L.) R.Br.	*PDBK2011-0894* (KB, KUS)	Kim et al. (2020b)	NC_046820 MN200391
*Gymnadenia crassinervis* Finet	unknown	unpublished	MW322684
*Habenaria aitchisonii* Rchb.f.	unknown	unpublished	MW316693
*Habenaria ciliolaris* Kraenzl.	China, Fujian, *YT-MTYFH* (FAFU)	Chen et al. (2019)	MN495954
*Habenaria crassilabia* Kraenzl.	*PDBK2018-1246* (KB, KUS)	Kim et al. (2020b) (listed as *Habenaria chejuensis* Y.N.Lee & K.Lee)	NC_046821 MN200392
*Habenaria cruciformis* Ohwi	South Korea, Gangwon, *GCU190036385* (GCU)	Kim et al. (2020a)	NC_059695 MT863537
*Habenaria dentata* (Sw.) Schltr.	China, Guangxi, *SFC-20201015012* (IBK)	Zhang et al. (2022)	OK012095
*Habenaria linearifolia* Maxim.	unknown	Kim et al. (2020a)	NC_059696 MT863538
*Habenaria pantlingiana* Kraenzl.	unknown	Lin et al. (2015) (as *Habenaria longidenticulata*, nom. nud.)	NC_026775 KJ524104
*Hemipilia gracilis* (Blume) Y.Tang, H.Peng & T.Yukawa	*PDBK2008-0404* (KB, KUS)	Kim et al. (2020b)	NC_046810 MN200376
*Hemipilia yajiangensis* G.W.Hu, Jia X.Yang & Q.F.Wang	China, Sichuan, *J.X. Yang, S. Peng, Y. Wang, S.X. Ding, J.J. Wang PS-00264* (HIB: 0188766, 0188768, 0188767, paratypes)	Yang et al. (2022)	NC_067080 OM009241
*Ophrys fusca* ssp. *iricolor* (Desf.) K.Richt.	Greece, Crete	Roma et al. (2018)	AP018716
*Ophrys insectifera* ssp. *aymoninii* Breistr.	France, Occitania	Bertrand et al. (2021)	MW309825
*Ophrys lutea* Cav.	France, Occitania	Bertrand et al. (2021)	NC_058525 MW309826
*Ophrys sphegodes* Mill.	Italy, Apulia	Roma et al. (2018)	AP018717
*Pecteilis radiata* (Thunb.) Raf.	*PDBK2015-1271*	Kim et al. (2020a)	NC_035834 KX871237
*Peristylus densus* (Lindl.) Santapau & Kapadia	*PDBK2018-1247* (KB, KUS)	Kim et al. (2020b) (listed as *Habenaria flagellifera* Makino)	NC_046801 MN200366
*Platanthera chlorantha* (Custer) Rchb.	Poland, *SG-13236* (UGDA)	Lallemand et al. (2019)	NC_044626 MK937914
*Platanthera japonica* (Thunb.) Lindl.	China, Shaanxi (WNU)	Dong et al. (2018)	NC_037440 MG925368
*Platanthera mandarinorum* Rchb.f.	*PDBK2013-0398* (KB, KUS)	Kim et al. (2020b)	NC_046805 MN200370
*Platanthera ussuriensis* (Regel) Maxim.	China, Liaoning, *B. Qu, X. Chen s.n.*	Han et al. (2022)	MN686021
*Satyrium nepalense* D.Don	China, Yunnan, *X.K. Ma 006* (FAFU)	Ma et al. (2019)	MN497244
*Vietorchis aurea* Aver. & Averyanova	Vietnam, Ninh Binh, *May Van Xinh MVX 261* (LE: LE01076723)	Cuc Phuong National Park, 200‒300 m, 05.2004	OQ318186*–OQ318196* (11 individual regions)
*Vietorchis furcata* Aver. & Nuraliev	Vietnam, Dak Lak, *M.S. Nuraliev 998* (LE: LE01076759)	Lak distr., Bong Krang munic., Chu Yang Sin national park, 12 km S of Krong Kmar village, forest, near small river, c. 1100 m, 12°23’41”N 108°20’55”E, 28.05.2014	OQ318871*
**Outgroups**			
*Goodyera pubescens* (Willd.) R.Br.	USA, Wisconsin, *JH170809001* (GCU)	Kim and Kim (2022)	NC_064116 OM314912
*Spiranthes sinensis* (Pers.) Ames	South Korea, Jeju, *TH200805012* (GCU)	Kim and Kim (2022)	NC_064118 OM314917

Asterisk denotes newly generated sequences.
