# Supplementary material for: Phylogenetic position and plastid genome structure of Vietorchis, a mycoheterotrophic genus of Orchidaceae (subtribe Orchidinae) endemic to Vietnam
Source: Front Plant Sci. 2024 May 24;15:1393225. doi: 10.3389/fpls.2024.1393225 (PMC11157612; doi:10.3389/fpls.2024.1393225)
Supplement: Supplementary file 1 [file DataSheet_1.pdf]

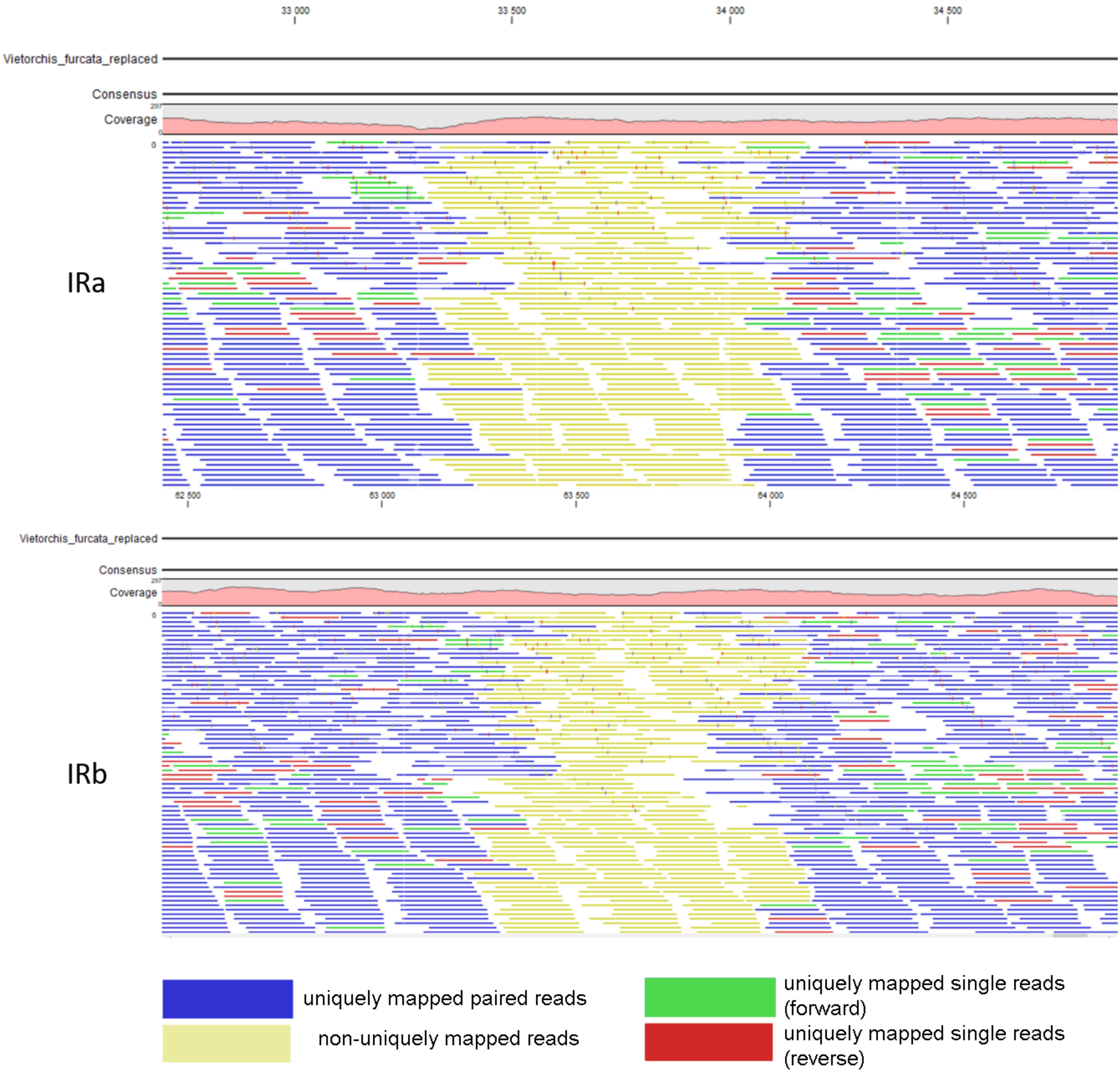

Supplementary Figure 1. Verification of the SC-IR borders based on raw read mapping. The mapping was performed and visualized using CLC Genomics Workbench. In the version of assembly submitted to the Genbank, the IRa is at the end of the sequence; therefore, for this check, we created a new assembly by placing the first 2000 nucleotides at the end, after the IRa.

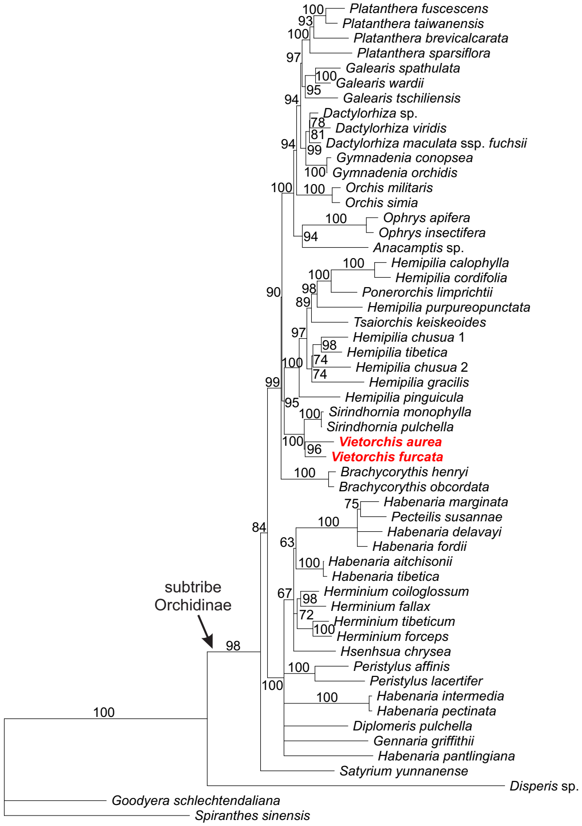

Supplementary Figure 2. Phylogenetic tree obtained from the ML analysis of the combined nuclear ITS+*Xdh* dataset. Numbers near branches indicate ultrafast bootstrap percentages.

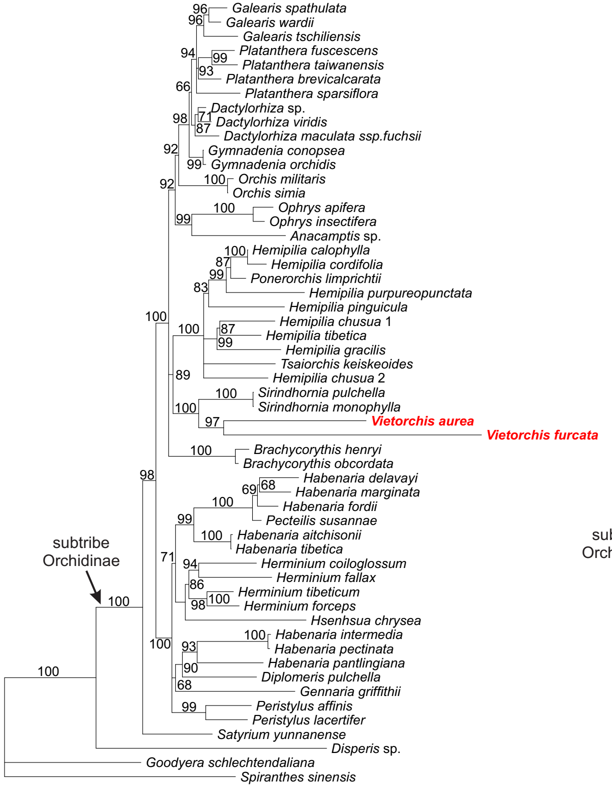

Supplementary Figure 3. Phylogenetic tree obtained from the ML analysis of the combined plastid *matK*+*psb*A-*trn*H dataset. Numbers near branches indicate ultrafast bootstrap percentages.

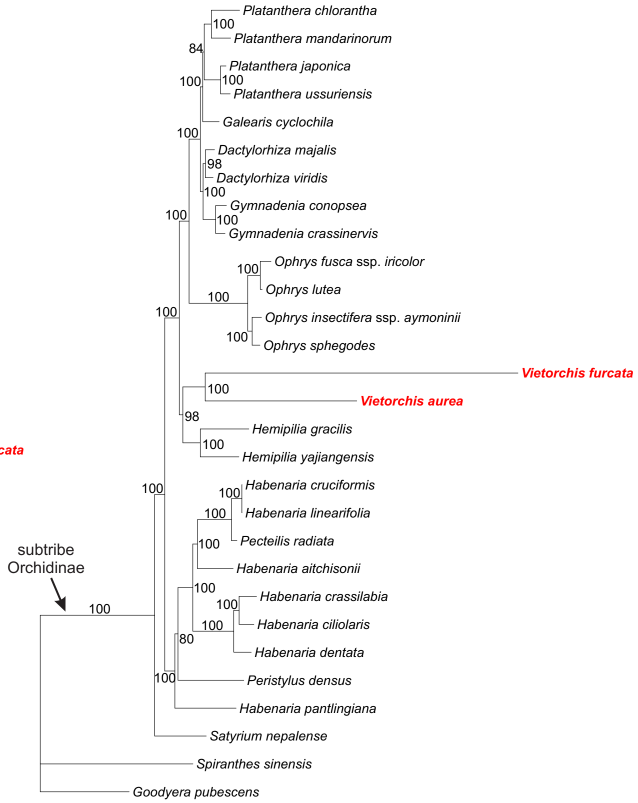

Supplementary Figure 4. Phylogenetic tree obtained from the ML analysis of the combined 29-gene plastid dataset. Numbers near branches indicate ultrafast bootstrap percentages.

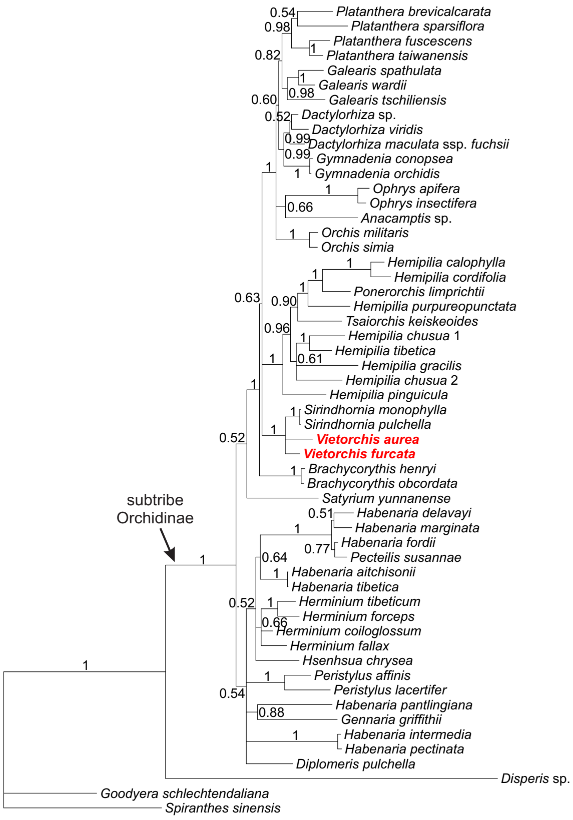

Supplementary Figure 5. Phylogenetic tree obtained from the Bayesian analysis of the ITS dataset.
Numbers near branches indicate posterior probabilities (PP).

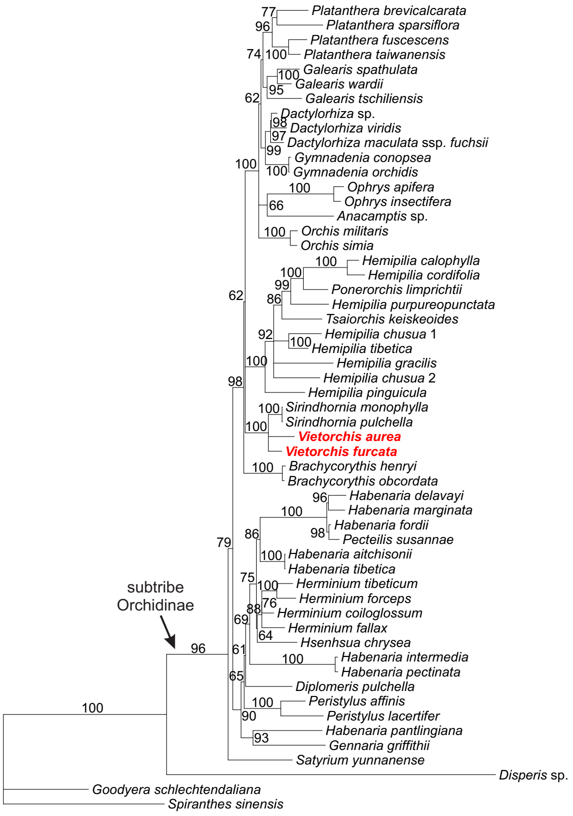

Supplementary Figure 6. Phylogenetic tree obtained from the ML analysis of the ITS dataset.
Numbers near branches indicate ultrafast bootstrap percentages.

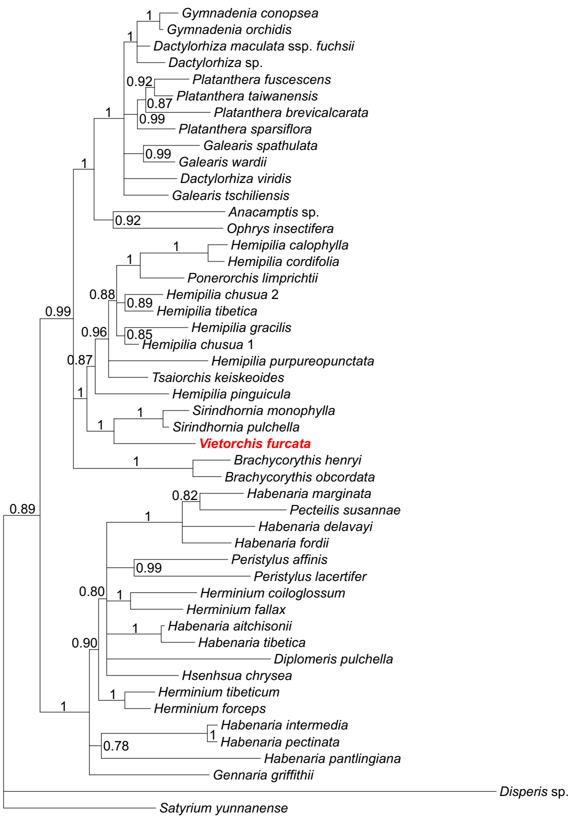

Supplementary Figure 7. Phylogenetic tree obtained from the Bayesian analysis of the *Xdh* dataset.
Numbers near branches indicate posterior probabilities (PP).

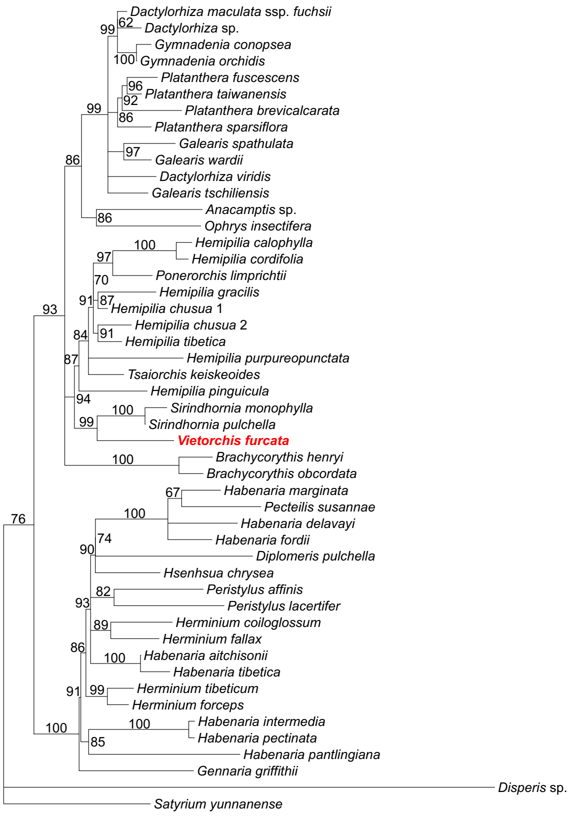

Supplementary Figure 8. Phylogenetic tree obtained from the ML analysis of the *Xdh* dataset.
Numbers near branches indicate ultrafast bootstrap percentages.

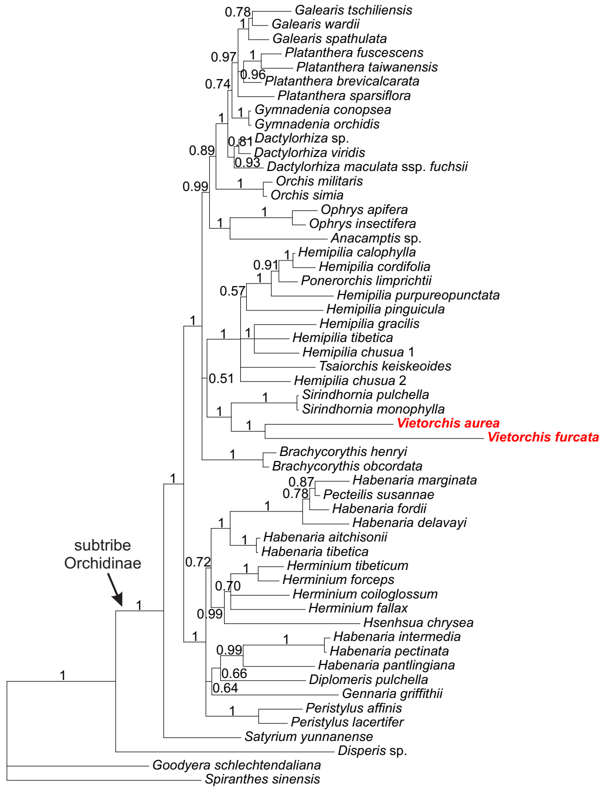

Supplementary Figure 9. Phylogenetic tree obtained from the Bayesian analysis of the *matK* dataset.
Numbers near branches indicate posterior probabilities (PP).

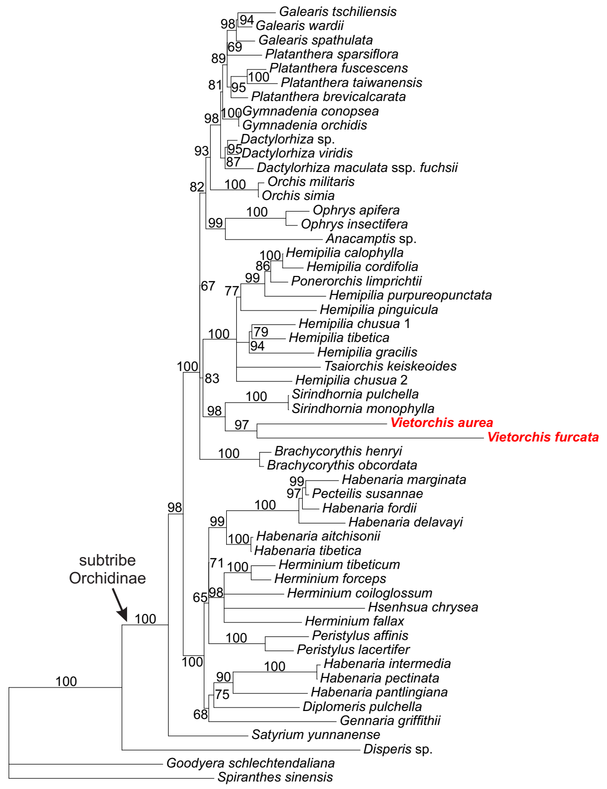

Supplementary Figure 10. Phylogenetic tree obtained from the ML analysis of the *matK* dataset.
Numbers near branches indicate ultrafast bootstrap percentages.

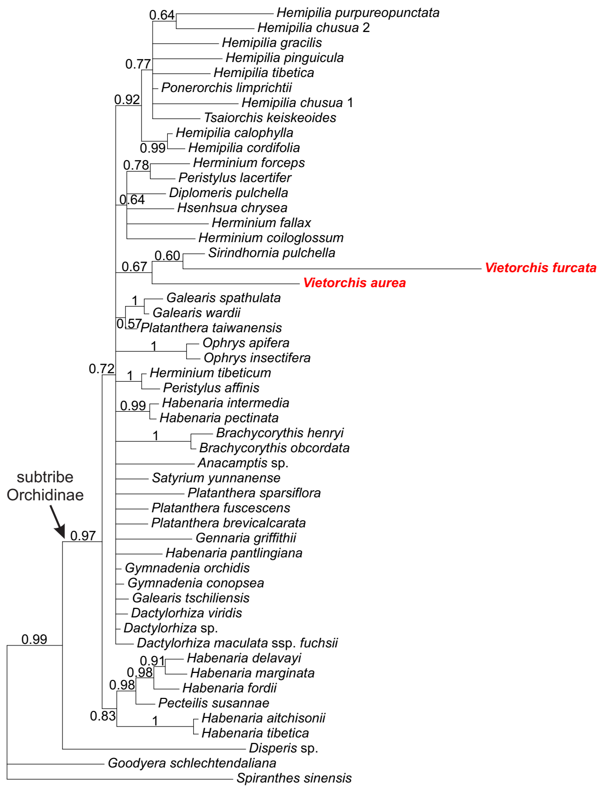

Supplementary Figure 11. Phylogenetic tree obtained from the Bayesian analysis of the *psb*A-*trn*H dataset.
Numbers near branches indicate posterior probabilities (PP).

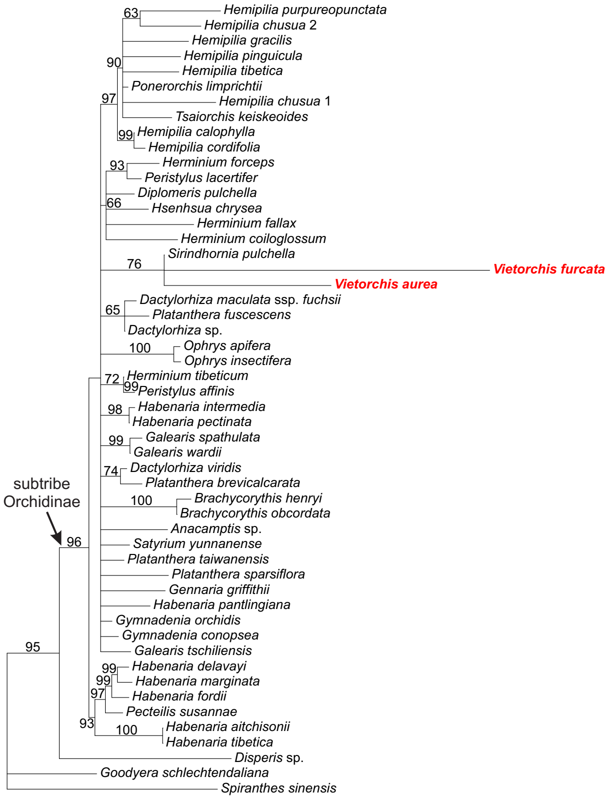

Supplementary Figure 12. Phylogenetic tree obtained from the ML analysis of the *psb*A-*trn*H dataset.
Numbers near branches indicate ultrafast bootstrap percentages.

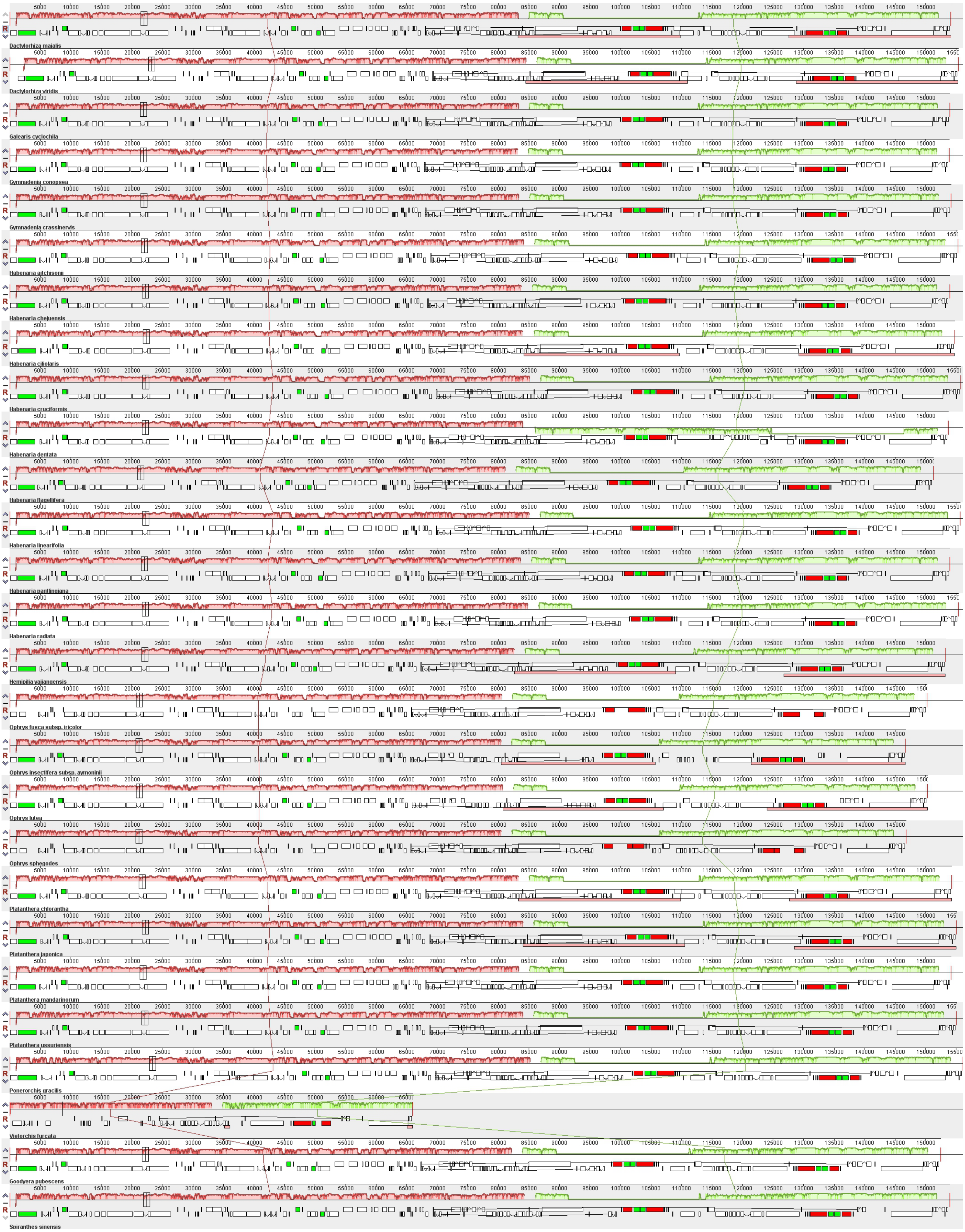

Supplementary Figure 13. Colinear blocks between the plastomes of *Vietorchis furcata* and the other studied species of the subtribe Orchidinae (and also *Goodyera pubescens* and *Spiranthes sinensis* used as an outgroup in phylogenetic reconstructions).

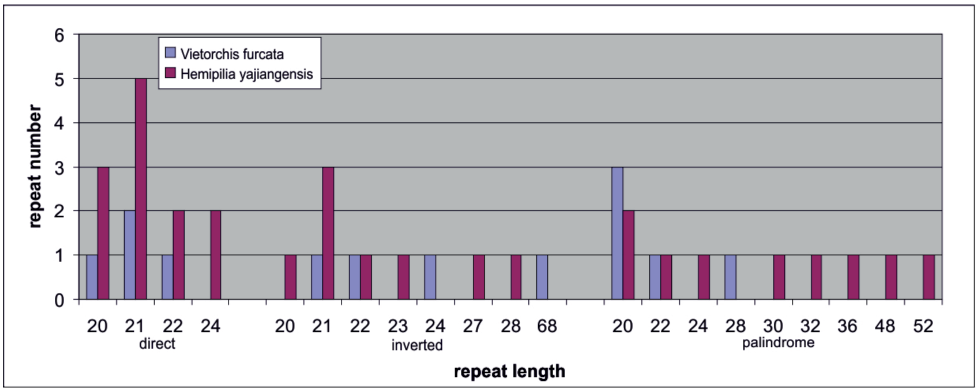

Supplementary Figure 14. Dispersed repeats in the plastomes of *Hemipilia yajiangensis* and *Vietorchis furcata*.
